# A Mindfulness-Based Lifestyle Intervention for Dementia Risk Reduction: Protocol for the My Healthy Brain Feasibility Randomized Controlled Trial

**DOI:** 10.2196/64149

**Published:** 2024-11-21

**Authors:** Ryan A Mace, Makenna E Law, Joshua E Cohen, Christine S Ritchie, Olivia I Okereke, Bettina B Hoeppner, Judson A Brewer, Stephen J Bartels, Ana-Maria Vranceanu, My Healthy Brain Team

**Affiliations:** 1 Center for Health Outcomes and Interdisciplinary Research (CHOIR) Department of Psychiatry Massachusetts General Hospital Boston, MA United States; 2 Harvard Medical School Boston, MA United States; 3 Mongan Institute Center for Aging and Serious Illness Division of Palliative Care and Geriatric Medicine Massachusetts General Hospital Boston, MA United States; 4 Channing Division of Network Medicine Department of Medicine Brigham and Women's Hospital and Harvard Medical School Boston, MA United States; 5 Department of Epidemiology Harvard T.H. Chan School of Public Health Boston, MA United States; 6 Department of Psychiatry Massachusetts General Hospital Boston, MA United States; 7 Health through Flourishing (HtF) Program Department of Psychiatry Massachusetts General Hospital Boston, MA United States; 8 Mindfulness Center Brown University School of Public Health Providence, MA United States; 9 Department of Psychiatry and Human Behavior Warren Alpert Medical School of Brown University Providence, MA United States; 10 Department of Medicine Massachusetts General Hospital Boston, MA United States; 11 The Mongan Institute Massachusetts General Hospital Boston, MA United States; 12 see Acknowledgments Milton, ON Canada

**Keywords:** lifestyle, cognitive decline, brain health, mindfulness, mind-body therapies, telemedicine, digital health, randomized clinical trial

## Abstract

**Background:**

Lifestyle behavior change and mindfulness have direct and synergistic effects on cognitive functioning and may prevent Alzheimer disease and Alzheimer disease–related dementias (AD/ADRD). We are iteratively developing and testing My Healthy Brain (MHB), the first mindfulness-based lifestyle group program targeting AD/ADRD risk factors in older adults with subjective cognitive decline. Our pilot studies (National Institutes of Health [NIH] stage 1A) have shown that MHB is feasible, acceptable, and associated with improvement in lifestyle behavior and cognitive outcomes.

**Objective:**

We will compare the feasibility of MHB versus an education control (health enhancement program [HEP]) in 50 older adults (aged ≥60 y) with subjective cognitive decline and AD/ADRD risk factors. In an NIH stage 1B randomized controlled trial (RCT), we will evaluate feasibility benchmarks, improvements in cognitive and lifestyle outcomes, and engagement of hypothesized mechanisms.

**Methods:**

We are recruiting through clinics, flyers, web-based research platforms, and community partnerships. Participants are randomized to MHB or the HEP, both delivered in telehealth groups over 8 weeks. MHB participants learn behavior modification and mindfulness skills to achieve individualized lifestyle goals. HEP participants receive lifestyle education and group support. Assessments are repeated after the intervention and at a 6-month follow-up. Our primary outcomes are feasibility, acceptability, appropriateness, credibility, satisfaction, and fidelity benchmarks. The secondary outcomes are cognitive function and lifestyle (physical activity, sleep, nutrition, alcohol and tobacco use, and mental and social activity) behaviors. Data analyses will include the proportion of MHB and HEP participants who meet each benchmark (primary outcome) and paired samples 2-tailed *t* tests, Cohen *d* effect sizes, and the minimal clinically important difference for each measure (secondary outcomes).

**Results:**

Recruitment began in January 2024. We received 225 inquiries. Of these 225 individuals, 40 (17.8%) were eligible. Of the 40 eligible participants, 21 (52.5%) were enrolled in 2 group cohorts, 17 (42.5%) were on hold for future group cohorts, and 2 (5%) withdrew before enrollment. All participants have completed before the intervention assessments. All cohort 1 participants (9/21, 43%) have completed either MHB or the HEP (≥6 of 8 sessions) and after the intervention assessments. The intervention for cohort 2 (12/21, 57%) is ongoing. Adherence rates for the Garmin Vivosmart 5 (128/147, 87.1% weeks) and daily surveys (105/122, 86.1% weeks) are high. No enrolled participants have dropped out. Enrollment is projected to be completed by December 2024.

**Conclusions:**

The RCT will inform the development of a larger efficacy RCT (NIH stage 2) of MHB versus the HEP in a more diverse sample of older adults, testing mechanisms of improvements through theoretically driven mediators and moderators. The integration of mindfulness with lifestyle behavior change in MHB has the potential to be an effective and sustainable approach for increasing the uptake of AD/ADRD risk reduction strategies among older adults.

**Trial Registration:**

ClinicalTrials.gov NCT05934136; https://www.clinicaltrials.gov/study/NCT05934136

**International Registered Report Identifier (IRRID):**

DERR1-10.2196/64149

## Introduction

### Background

Alzheimer disease and Alzheimer disease–related dementias (AD/ADRD) are debilitating neurodegenerative disorders that impair cognitive and daily functioning [1]. An estimated 50 million people are living with AD/ADRD worldwide, with 10 million new cases occurring annually [2-4]. AD/ADRD places an immense burden on individuals, families, and health care systems. The economic toll of family and unpaid caregiving for AD/ADRD was US $346.6 billion in 2023 [5]. In the early stages of AD/ADRD, approximately half of adults aged ≥65 years perceive subjective cognitive decline (SCD) [6,7] in memory or other cognitive domains before neurodegeneration can be detected through cognitive testing [8,9]. SCD is associated with a greater likelihood of underlying AD/ADRD biomarker pathology and increased risk for future cognitive decline and AD/ADRD [10-13]. This preclinical stage is a critical window to engage older adults in preventive interventions that aim to modify AD/ADRD risk factors.

Growing research indicates that multiple lifestyle behaviors are important for AD/ADRD prevention [14-21]. In 2020, the Lancet Commission on Dementia identified 12 modifiable risk factors accounting for 40% of AD/ADRD cases worldwide: less education, hypertension, hearing impairment, smoking, obesity, depression, physical inactivity, diabetes, low social contact, excessive alcohol consumption, a history of head injury, and exposure to air pollution [22]. The commission also highlighted the need for further research on additional lifestyle risk factors such as poor sleep [23-25], diet [23,26], and mental activity [27,28], which may influence AD/ADRD risk but have shown mixed findings [29]. Multidomain trials targeting modifiable lifestyle risk factors identified in the *Lancet* report have demonstrated the potential to improve cognition among older adults with early cognitive decline [30,31]. However, not all multidomain trials have reported positive results [32-34]. Trials have also failed to modify behaviors [33], or they have inconsistently reported lifestyle outcomes [30]. Complex and time-intensive intervention designs hindered participant adherence [35,36] and limit future implementation. Additional research is needed to develop more effective and practical interventions that promote sustained engagement and adherence to AD/ADRD prevention strategies among older adults.

Mindfulness practices may help address challenges in modifying lifestyle behaviors and offer direct brain health benefits. Mindfulness is commonly defined as the practice of nonjudgmental awareness of the present moment [37]. Mindful self-regulation theory suggests that mindfulness involves several self-regulation processes (eg, emotion regulation, cognitive control, and self-monitoring) that enhance one’s ability to cope with urges (eg, overeating or avoiding exercise) and make healthier lifestyle choices [38-40]. Mindfulness practice is positively associated with measures of enhanced brain structure [41-43] and cognitive function [41,44-48]. In addition, mindfulness is associated with reductions in psychological symptoms, such as depression, anxiety, and attitudes and worries regarding AD/ADRD [49-52], which are independent risk factors for AD/ADRD [53-56] and an inactive lifestyle [57]. Mindfulness is feasible and acceptable for older adults, including individuals with SCD [38,52,58]. Prior research suggests that cognitive benefits associated with mindfulness can be observed with brief practice [59-61], suggesting that mindfulness is amenable to time-limited interventions. Despite these advantages, mindfulness has been overlooked in behavior modification AD/ADRD prevention interventions.

**Figure 1:**
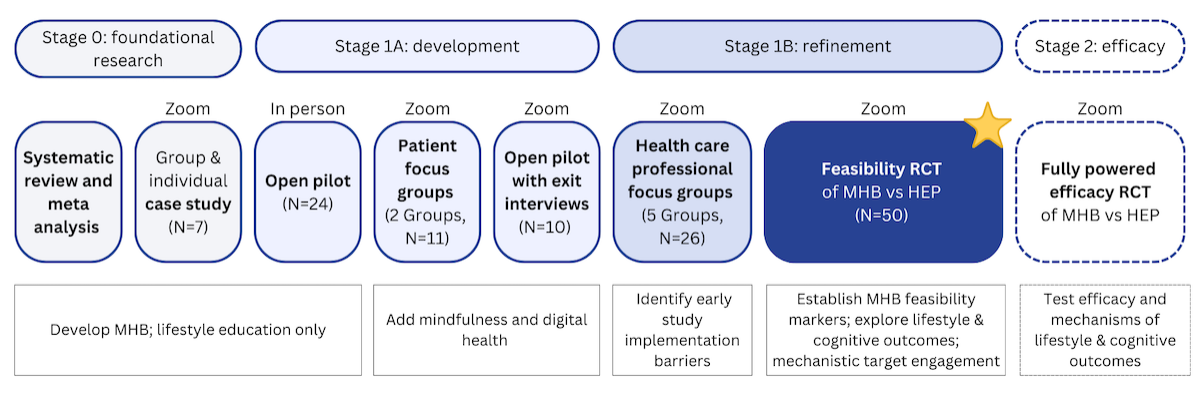
Iterative development of My Healthy Brain (MHB) following the National Institutes of Health Stage Model. The current feasibility randomized controlled trial (RCT; starred box) will inform an efficacy RCT (dashed box). HEP: health enhancement program.

Our interdisciplinary team has developed the first group mindfulness-based lifestyle intervention (My Healthy Brain [MHB]) that aims to modify early risk for AD/ADRD. We conducted a series of preliminary studies to develop MHB following the National Institutes of Health (NIH) Stage Model [62], an iterative framework for guiding behavioral intervention development and testing, from pilot studies to implementation and dissemination (Figure 1). First, our systematic review and meta-analysis (we analyzed 79 studies with 9233 participants) found moderate- to high-quality evidence that mindfulness-based interventions were associated with significant improvements in multiple lifestyle behaviors linked to brain health, including sleep, physical activity, alcohol use, and tobacco cessation [63]. Second, we conducted iterative studies of older adults with AD/ADRD risk factors to determine the best delivery modality and strategies to incorporate mindfulness into lifestyle behaviors. Preliminary studies included (1) an in-person clinical pilot (N=24) [64], (2) a telehealth group case series (N=7) [65], and (3) a mixed methods study to adapt the program via qualitative focus groups (N=11) and conduct a feasibility pilot with exit interviews (N=10) [66]. MHB met benchmarks set a priori for feasibility, credibility, satisfaction, and safety (there were no adverse events). We observed preliminary improvements (moderate to large effects) in subjective and objective measures of cognitive function, physical activity, sleep, and proposed mechanisms. Exit interviews confirmed the satisfaction with mindfulness and feasibility with technologies to support behavior change (monitoring steps via an activity watch) and remote delivery (Zoom; Zoom Video Communications, Inc). Finally, we conducted a qualitative study with health care professionals caring for older patients (N=26) to address barriers to implementing study procedures (eg, recruitment, enrollment, and retention) and maximizing diversity to prepare for the first feasibility randomized controlled trial (RCT) of MHB [67].

### Objectives

Building upon our preliminary studies, we report on the improved protocol and initial launch of the first feasibility RCT of MHB (NIH stage 1B). We are enrolling 50 older adults (aged ≥60 y) with SCD and modifiable AD/ADRD lifestyle risk factors. Participants are randomized to MHB or a time- and attention-matched education control (health enhancement program [HEP]) [68,69], both delivered in telehealth groups over 8 weeks. The primary aim is to assess the feasibility, acceptability, appropriateness, credibility, satisfaction, and fidelity of MHB against established Go–No-Go benchmarks [64,70-74]. The secondary aim is to investigate preliminary improvements in cognitive and lifestyle outcomes and engagement in proposed mechanisms. The results will inform the first efficacy RCT of MHB and mechanistic testing of cognitive and lifestyle outcomes.

## Methods

### Overview

**Figure 2:**
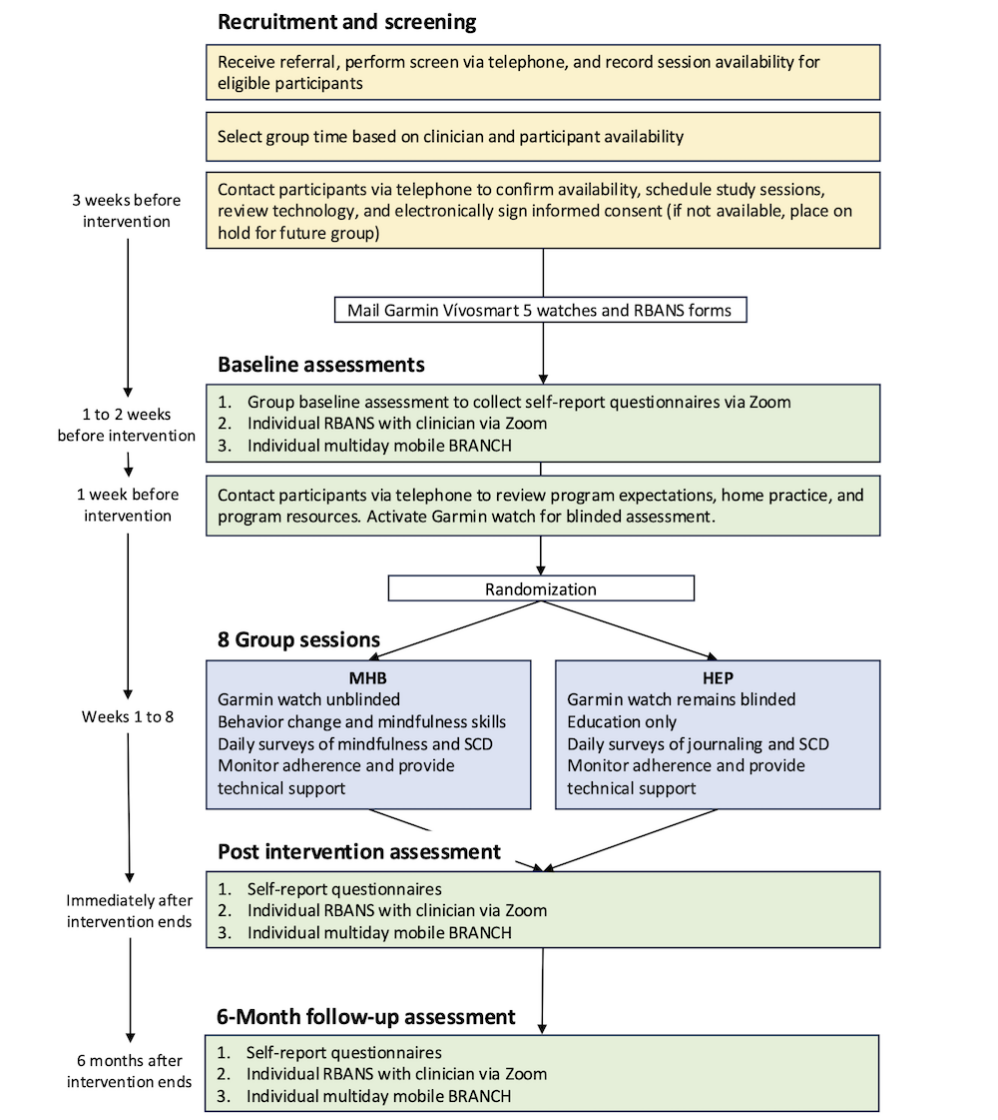
Study flow and timeline. Color key: yellow=recruitment, green=assessment, and blue=programs. BRANCH: Boston Remote Assessment for Neurocognitive Health; Garmin: Garmin Vivosmart 5 watch; HEP: health enhancement program; MHB: My Healthy Brain; RBANS: Repeatable Battery for the Assessment of Neuropsychological Status; SCD: subjective cognitive decline.

We are conducting a virtual, single-blind, feasibility RCT of the MHB intervention versus the HEP [68,69] education control (N=50). Our study is consistent with the research objectives and activities specified in stage 1B of the NIH Stage Model [62]. NIH stage 1 focuses on intervention development, testing, refinement, and modification. NIH stage 1B activities emphasize the refinement of intervention and training materials, feasibility and pilot testing, and early attention to implementation. As such, our study is designed to confirm the feasibility of MHB and assess engagement in intervention outcomes and hypothesized mechanisms before conducting a fully powered efficacy RCT [75-77]. All procedures described herein are conducted fully remotely, allowing for both local and national recruitment. We followed the National Council on Aging guidelines for using technology with older adults [78] and our established digital health trials [66,70,74,79-92]. Figure 2 presents the study flow.

We preregistered our RCT on ClinicalTrials.gov (NCT05934136) before enrolling the first participant.

### Ethical Considerations

The Mass General Brigham (MGB) institutional review board (IRB) approved all study procedures (2023P001770). All participants review and sign written informed consent with a clinical research assistant (RA) before completing study procedures. Consent informs participants that, with any group-based intervention, there may be confidentiality and privacy risks. To minimize these risks, we discuss the importance of confidentiality at the start of each group; request that participants attend the session from a private location; and all data are deidentified, maintained in a secured location, and only accessed by IRB-approved members of the research team. Participants are compensated up to US $220: US $30 for before the intervention assessments, US $60 for after the intervention assessments, US $90 for 6-month follow-up assessments, with an additional US $40 for 7 out of 7 valid days of Garmin Vivosmart 5 wear during the before the intervention assessment period. This compensation strategy is meant to optimize participant engagement and motivation for 6-month follow-up assessments.

### Participants

Participants are older adults (aged ≥60) who are at early risk for AD/ADRD as determined by the presence of SCD [8], have no cognitive impairment (Telephone Interview for Cognitive Status score >30) [93], and have modifiable AD/ADRD risk factors (Cardiovascular Risk Factors, Aging, and Incidence of Dementia [CAIDE] score ≥6) [94,95]. Textbox 1 shows our full inclusion and exclusion criteria and rationales, which we have refined through our previous pilot studies [64-66]. These eligibility criteria were informed by similar lifestyle AD/ADRD prevention trials [30,31,35] and behavioral interventions for older adults [74,81,83,92,96].

**Study inclusion and exclusion criteria with rationales.**

**Inclusion criteria and brief rationales**

1. Aged ≥60 years: study population
2. Subjective cognitive decline (SCD; eg, forgetting information, getting lost, and repeating oneself): SCD Initiative criteria [8]
3. Cardiovascular Risk Factors, Aging, and Incidence of Dementia score ≥6 [94,95]: modifiable Alzheimer disease and Alzheimer disease–related dementias (AD/ADRD) risk factors [30,35]
4. Telephone Interview for Cognitive Status score >30 [93]: absence of AD/ADRD that would prevent meaningful engagement
5. Functional Assessment Questionnaire score <9 [97,98]: functional independence and ability to participate meaningfully
6. English fluency and literacy: data validity (all measures are validated for use in English) and delivery modality
7. Ability and willingness to participate via live video in group session: delivery modality
8. No self-reported safety issues with initiating lifestyle changes during the study: safety of participants and validity

**Exclusion criteria and brief rationales**

1. Mild cognitive impairment, AD/ADRD, or other neurodegenerative disease: study confound (intervention targets early AD/ADRD risk; ability to participate meaningfully)
2. Psychotropic medication (eg, antidepressant) change in <3 months: study confound
3. Psychosis, uncontrolled bipolar disorder, or uncontrolled substance dependence or abuse: study confound (safety of participants; treatment confound; can affect participants’ answers)
4. Current self-report of suicidal ideation: participant safety
5. Serious medical illness expected to worsen in 6 months (eg, cancer): study confound (serious medical illness may act as a third variable)
6. Use of an activity watch to track physical activity or sleep in <3 months and unwillingness to stop using it for the duration of the program: treatment confound
7. Mindfulness practice lasting >45 minutes per week or cognitive behavioral therapy in <3 months: treatment confound
8. Self-reported step count >5000 per day or ≥30 minutes of exercise per day: study population (intervention targets increased activity among sedentary older adults)

### Recruitment and Enrollment

Our recruitment strategy incorporates a combination of hospital partnerships, community outreach, and internet-based recruitment methods. Through our preliminary studies, we developed partnerships with several MGB clinics that are directly involved in the care of older patients with SCD. The clinics represent expertise in geriatrics, geriatric psychiatry, neurology, neuropsychology, memory care, and integrative medicine. To promote the study within our hospital, we have engaged clinic leadership, presented in team meetings, and distributed our IRB-approved flyer to providers and clinic waiting areas. The flyer contains study contact information and a QR code to an eligibility self-screener. In addition, patients can contact the study through a hospital web-based recruitment platform (MGB Rally). We aim to maximize the inclusion of older adults from diverse and underserved backgrounds to address disparities in AD/ADRD [99,100] and underrepresentation in prevention clinical trials [101,102]. First, we have proactively formed collaborations with MGB clinics that serve more diverse patients. Second, we have engaged community organizations and Councils on Aging (commonly known as “senior centers”) in Massachusetts. Community outreach consists of engaging community leadership, disseminating recruitment materials, and conducting public events (eg, networking nights and educational talks). We are specifically targeting outreach to underresourced communities of older adults in Massachusetts who face significant health disparities and heightened risk factors for AD/ADRD [103]. Third, we have consulted with our center’s Community Engagement Core on evidence-based recruitment strategies [104]. Fourth and last, we have expanded our outreach nationally via social media platforms, web-based study postings (eg, ClinicalTrials.gov and Alzheimers.gov), and articles and presentations developed for public audiences.

### Screening and Enrollment

The RA logs all referral information in a standardized REDCap (Research Electronic Data Capture; Vanderbilt University) [105] database. Participants have the option to complete an initial screening survey independently or by telephone with the RA. The RA contacts all potentially eligible participants to answer questions about the study, collect availability for groups, and assess technology access and readiness. Individuals who do not meet study criteria are offered resource sheets developed for older adults with SCD. The principal investigator (RAM) reviews all eligibility decisions and consults with the multidisciplinary study team if needed. After group times have been set, the RA meets with participants individually over Zoom to schedule the before the intervention assessments, review study technology, and obtain electronic informed consent via REDCap. The RA uses secure email to send confirmation and Zoom links for the before the intervention assessments.

### Assessments

#### Overview

Tables 1 and 2 detail the assessments and the frequency of data collection. All assessment data are stored in a REDCap database. An RA blinded to randomization reviews all measures for missing data, errors, or invalid responses.

**Table 1:** Primary outcomes.

Construct and measure			Scoring		Criteria		Time point
**Feasibility**							
	Recruitment and enrollment	Percentage of individuals who participate and enroll from the total contacted		≥70%=good, ≥80%=excellent		Before the intervention	
	Outcome assessments	Percentage of participants with no missing outcome assessment data		≥70%=good, ≥80%=excellent		Before the intervention, after the intervention, and at 6-month follow-up	
	Garmin Vivosmart 5 watch	Percentage of participants who wore the watch at least 5 out of 7 days per week [106,107] for at least 10 hours a day [71]		≥70%=good, ≥80%=excellent		After the intervention	
	Daily surveys	Percentage of participants who completed at least 5 out of 7 daily surveys during the program		≥70%=good, ≥80%=excellent		After the intervention	
**Acceptability**							
	Satisfaction	Client Satisfaction Questionnaire [108] assesses patient satisfaction with the program; percentage of participants with scores (minimum=3, maximum=12) above the scale’s midpoint		≥70%=good, ≥80%=excellent		After the intervention	
	Program attendance	Percentage of participants who attend ≥6 out of 8 sessions		≥70%=good, ≥80%=excellent		After the intervention	
	Perceived improvements	Modified Patient Global Impression of Change [109] asks participants about perceived improvements in cognitive function, lifestyle, and emotional well-being outcomes; percentage of participants who report improvement in each outcome domain (ranging from 1=*very much worse* to 7=*very much improved*)		≥70%=good, ≥80%=excellent		After the intervention and at 6-month follow-up	
**Appropriateness**							
	Credibility and expectancy	Credibility and Expectancy Questionnaire [110] assesses participant perceptions of the treatment as believable, convincing, and logical; percentage of participants with scores (minimum=3, maximum=27) above the scale’s midpoint		≥70%=good, ≥80%=excellent		Before the intervention	
**Fidelity**							
	Therapist fidelity	Percentage of sessions in which the study clinician completed an audio recording, progress note, and checklist with 100% of the content delivered (confirmed by blinded RA independently coding a random 10% of audio-recorded sessions)		≥75%=good, 90%=excellent		Study completion	
	Study procedures	Fidelity of staff to the study procedures as calculated by the frequency of protocol deviations		<5 deviations=good, 0 deviations=excellent		Study completion	
Patient safety			Number of self-reported adverse events		Mild in ≤10% of participants=good, none=excellent		Study completion

**Table 2:** Study measures and constructs. Demographic and clinical characteristics are assessed before the intervention. All other measures are assessed before the intervention, after the intervention, and at 6-month follow-up.

Measure and description		Scoring	Psychometric evidence
**Demographic characteristics**			
	Age, gender, biological sex, race, ethnicity, education, income, occupation, marital status, living situation, and languages spoken	—^a^	—
**Clinical characteristics**			
	Mental health history, medical history, height, weight, Lifestyle for Brain Health (LIBRA) score [111-116], SCD^b^ diagnosis, medications and supplements taken for memory	—	—
**Cognitive function**			
	Cognitive Function Index [117], a 14-item measure assessing self-report of SCD across daily functions	1 (*yes*) to 0 (*no*) cognitive changes compared to 1 year ago; higher total scores (minimum=0, maximum=14) indicate greater SCD complaints	Adequate internal consistency and validity for older adults [118-120]
	Repeatable Battery for the Assessment of Neuropsychological Status (RBANS) [121], a comprehensive assessment that includes measures of visual, verbal, and numeric associative memory properties and provides a measure of global cognition	Higher *z* and index scores (minimum=0, maximum=160) indicate greater global and domain-specific cognitive functioning	Clinically valid [121] and high internal reliability among older adults [122]
**Physical activity**			
	PROMIS^c^ Physical Function [123]*,* an 8-item self-report of daily physical functioning	5-point scale ranging from 1=*unable to do* to 5=*without any difficulty*; higher T-scores indicate greater physical function and lower disability	Acceptable construct validity for older adults, sensitive to change during intervention studies, and excellent internal reliability [123,124]
	Godin Leisure-Time Exercise Questionnaire [125], a 3-item self-report of the frequency of engagement in light, moderate, and vigorous physical activity	Number of times per week engaged in activity; higher scores indicate greater frequency of physical activity for each intensity level	Good construct validity [126] and relatively reliable [127]
	Change in step count, measured via the Garmin Vivosmart 5 [128] (change in average step count during the 7 days preceding before the intervention assessment, throughout the intervention period, and 7 days at 6-month follow-up)	Higher step count totals indicate greater physical activity levels (walking)	Low actigraphy-measured step count is correlated with AD/ADRD^d^ risk and cognitive decline [129]; MCID^e^=600 to 1100 steps [130]
**Sleep**			
	Pittsburgh Sleep Quality Index [131], a 9-item self-report of sleep patterns and overall quality	Combination of 4-point scale ranging from 1=*not during the past month, very good* to 4=*≥3 times per week, very bad* and open responses; total scores range from 0 to 21, with a score of ≥5 indicating clinically significant sleep disturbance	Prior research supports the Pittsburgh Sleep Quality Index in older adults; high test-retest reliability, fair internal reliability [132], and good validity [133]
	Change in total sleep time, measured in minutes via the Garmin Vivosmart 5 [128] (change in total sleep time during the 7 days preceding before the intervention assessment, throughout the intervention period, and 7 days at 6-month follow-up)	Higher total minutes indicate greater sleep time	Poor actigraphy-measured sleep is correlated with AD/ADRD risk and cognitive decline [134]; MCID=40-minute increase [135]
**Mediterranean diet**			
	Mediterranean Eating Pattern for Americans Screener [131,136], a 16-item self-report of adherence to Mediterranean dietary recommendations	Participants earn 1 point for each food within the recommended serving size (range=0-16); higher total scores (minimum=0, maximum=21) indicate greater adherence to the Mediterranean diet	Poor internal reliability [131,136]; however, brief self-reports of the Mediterranean diet are limited
**Alcohol and tobacco use**			
	PROMIS Alcohol Use [137]*,* a 7-item self-report of at-risk drinking	5-point scale ranging from 1=*never* to 5=*almost always*; higher T-scores indicate greater problematic alcohol use	High convergent validity [137] and modest test-retest reliability [138]
	CDC^f^ Behavioral Risk Factor Surveillance System questionnaire concerning tobacco use [139]*,* a 2-item self-report of tobacco use history and current frequency of use of 6 common tobacco products	5-point scale ranging from 1=*less than once a month* to 5=*daily or almost daily*; higher scores indicate greater tobacco use	—
**Social functioning**			
	PROMIS Loneliness [140], a 5-item self-report of perceived loneliness	5-point scale ranging from 1=*never* to 5=*always*; higher T-scores indicate greater perceived loneliness	Good internal and test-retest reliability [141]
	Social Engagement and Activities Questionnaire [142], a 10-item self-report of general and social-group activities	6-point scale ranging from 1=*not at all* to 6=*every day*; higher total scores (minimum=0, maximum=50) indicate greater participation in socially engaging activities	High convergent validity for older adults [142]
	PROMIS Satisfaction with Social Roles and Activities [143], an 8-item self-report of satisfaction with ability to perform social activities and meet social needs	5-point scale ranging from 1=*not at all* to 5=*very much*; higher T-scores indicate greater satisfaction with social roles and activities	High reliability and acceptable item-total correlations [144]
**Mental activity**			
	Memory Compensation Questionnaire [145], a 44-item measure assessing the use of cognitive compensatory strategies for actual or perceived memory loss; the external and internal subscales (12 items) relevant to the MHB^g^ program are extracted	5-point scale ranging from 1=*never* to 5=*always*; higher total scores (minimum=0, maximum=65) indicate greater use of external and internal memory compensation strategies	Good internal validity among older adults [145]
	Measure of cognitive activities (adapted from Geda et al [146]), a 10-item self-report measure that assesses the extent to which an individual engaged in mentally stimulating and social activities over the past week	5-point scale ranging from 1=*never* to 5=*every day*; higher total scores (minimum=0, maximum=10) indicate greater participation in cognitively stimulating activities	Good test-retest reliability [147] and adequate construct validity [148]
**Depression and anxiety**			
	PROMIS Depression [149]*,* a 4-item measure assessing negative mood, views of self, engagement in daily living, and social components	5-point scale for depressive symptoms ranging from 1=*never* to 5=*always*; higher T-scores indicate greater depression	High reliability estimates among diverse older adults and clinically valid [150,151]
	PROMIS Anxiety [149]*,* a 4-item measure assessing fear, worry, hyperarousal, and somatic symptoms	5-point scale for anxiety symptoms ranging from 1=*never* to 5=*always*; higher T-scores indicate greater anxiety	High reliability estimates among diverse older adults [150] and strong validity [152]
**Mindfulness**			
	Applied Mindfulness Process Scale [153]*,* a 15-item instrument that measures the frequency of mindfulness practice	5-point scale ranging from 1=*never* to 5=*almost always*; higher total scores (minimum=0, maximum=60) indicate greater use of daily mindfulness activities	Strong internal consistency reliability and item-total reliability [153]
**Self-regulation**			
	Emotion Regulation Questionnaire [154], a 10-item self-report of emotion regulation strategies, both in how emotions are felt and expressed	7-point scale ranging from 1=*strongly disagree* to 7=*strongly agree*; higher total scores (range 10-70) indicate greater tendency to regulate emotions using cognitive reappraisal and expressive suppression strategies	Fair internal reliability among older adults [155] and strong validity [156]
	Cognitive Control and Flexibility Questionnaire [157], an 18-item self-report of control over unwanted experiences	4-point scale ranging from 1=*seldom or never* to 4=*almost always*; higher total scores (minimum=13, maximum=52) indicate greater daily use of cognitive control and flexibility	Excellent internal consistency and good construct validity in a community sample [157]
**Attitudes to change AD/ADRD behaviors**			
	Motivation to Change Lifestyle and Health Behaviours for Dementia Risk Reduction scale [158], a 27-item self-report of attitudes to change behaviors to prevent AD/ADRD	5-point Likert scale ranging from 1=*strongly disagree* to 5=*strongly agree*; higher total scores (minimum=27, maximum=135) indicate greater motivation to alter lifestyle factors and health behaviors to reduce risk of dementia	Moderate to high internal reliability and test-retest reliability in a sample of older adults without dementia [158]
**Exploratory assessments**			
	Multiday Boston Remote Assessment for Neurocognitive Health [159], a 7-day, mobile, self-administered assessment measuring paired associative learning based on everyday objects (signs, groceries, and faces)	Digit-Signs Test (correct number of street sign–number pairings identified), Groceries Prices Test (correct number of grocery-price pairings identified), Face-Name Test (average of correct responses from 2 face-name pairings: first letter name recall and full name recall); higher scores on learning curve (scored over 7 days) indicate better performance (minimum=0, maximum=1)	Good test-retest reliability of the learning curves and excellent reliability between participants’ 2 composite learning curves [160]
	Daily surveys of mindfulness practice (MHB), journaling (HEP^h^), and SCD symptoms (both groups)	Daily mindfulness completed (*yes* or *no*), skills practiced (categorical: all that apply), and time spent practicing these skills (total minutes) in MHB; minutes spent journaling in the HEP [161]; SCD symptoms measured using an 11-point Likert scale ranging from 0=*bad: my thinking is very difficult or slow* to 10=*good: my thinking is sharp and quick* [162,163]	—

^a^Not applicable.

^b^SCD: subjective cognitive decline.

^c^PROMIS: Patient-Reported Outcomes Measurement Information System.

^d^AD/ADRD: Alzheimer disease and Alzheimer disease–related dementias.

^e^MCID: minimal clinically important difference.

^f^CDC: Centers for Disease Control and Prevention.

^g^MHB: My Healthy Brain.

^h^HEP: health enhancement program.

#### Benchmarks

Consistent with NIH stage 1B [62] and pilot study guidelines [76,77], our primary outcomes are a priori markers for feasibility, acceptability, appropriateness, credibility, satisfaction, and fidelity. We set Go–No-Go benchmarks based on similar pilot studies of technology-enabled behavioral interventions for older adult populations [64,70-74].

#### Self-Reported Outcomes

Our selection of self-reported outcomes was guided by our conceptual model (refer to the MHB Intervention subsection), our stage 1 preliminary studies [64-66], and similar lifestyle trials [30,31]. Self-reported outcomes are collected before the intervention, after the intervention, and at the 6-month follow-up during a 60-minute Zoom session. Participants receive a secure link to the self-reported outcomes survey via email from the RA. To aid focus, the RA mutes all participants in the group Zoom session. Participants can use the Zoom hand-raise function or temporarily unmute themselves to seek technical support or ask questions. This system has been used to remotely collect self-reported outcomes for multiple trials of older adults with cognitive impairment [74,83].

#### Repeatable Battery for the Assessment of Neuropsychological Status

The Repeatable Battery for the Assessment of Neuropsychological Status (RBANS) is a comprehensive assessment battery of multidomain cognitive performance [121]. We replaced the Montreal Cognitive Assessment used in our preliminary studies with the RBANS to improve sensitivity to preclinical cognitive changes among older adults with SCD [164-166]. Additional advantages of the RBANS include (1) a total of 4 forms available for repeated assessments to reduce practice effects [167], (2) a comprehensive assessment of 12 subtests in 20 to 30 minutes, (3) strong psychometric properties [168], (4) established telepractice guidelines [169], and (5) administration protocols developed by our team based on similar virtual trials [83].

Trained clinicians administer the RBANS to individual participants via Zoom following a standardized protocol informed by telepractice guidelines [169]. Participants are mailed all testing materials (eg, figure drawing and coding sheets) in advance. Before testing, clinicians ensure that the participants’ environment is optimal (quiet, private, no distractions, writing surface, adequate lighting, etc), internet connection is stable, and audio and video are clear. Participants without access to a large-screen device are provided an iPad to ensure clear visibility of the testing stimuli. Participants mail back their written materials for scoring using a prepaid envelope.

#### Garmin Vivosmart 5 Watch

We selected the Garmin Vivosmart 5 to replace the ActiGraph GT9X. Our preliminary studies revealed that the ActiGraph GT9X is cost prohibitive and frequently had technical support issues that resulted in interrupted data collection and study operations. Additional participant and scientific advantages of the Garmin Vivosmart 5 include (1) its commercial availability and user-friendly design, which promote future scalability; (2) the ability to conduct blinded assessments at study end points by selecting watch faces that do not display step count data; and (3) its reliability and validity for passive monitoring of physical activity and sleep among older adults [170-174]. Our testing showed that step counts measured using the Garmin Vivosmart 5 were internally consistent and produced similar estimates to another widely used device (Fitbit).

The Garmin Vivosmart 5 serves 2 purposes in the RCT. First, in conjunction with the Garmin Connect app, it provides self-monitoring tools (eg, real-time step counting and “move” notifications) to promote engagement in physical activity and sleep [171]. Second, it provides objective, continuous, and passive data to capture within-person changes [175,176] in these lifestyle behaviors during the intervention [177,178]. Wearable devices are both feasible and acceptable for older adults and provide valid measurements of physical activity and sleep [179].

The Garmin Vivosmart 5 is mailed to participants 1 to 2 weeks before the intervention phase, and they set up the watch with the RA during an individual Zoom session. The RA pairs the watch with the participant’s mobile phone and reviews basic instructions (how to wear, charge, and sync). Participants are instructed to wear the Garmin Vivosmart 5 on their nondominant hand for 24 hours per day (except when briefly removing it for charging) to track steps and sleep. The watch is waterproof and can last up to 7 days per charge. Participants sync their data using the Garmin Connect app daily and charge the watch regularly. The RA turns off self-monitoring features (step count and sleep notifications) for blinded before the intervention assessments in both MHB and HEP groups. After the before the intervention assessments are complete, the RA turns on the self-monitoring features in the MHB group only because activity reinforcement is a core component of the technology-enhanced intervention.

Garmin Vivosmart 5 adherence is defined as ≥10 hours of valid wear time based on prior research [180-183]. Wear time is calculated in relation to heart rate by Fitabase [184]. The RA monitors Fitabase and contacts participants after 48 hours of Garmin Vivosmart 5 nonadherence to provide technical support. We estimate a truncated average 7-day step count and sleep data for each participant, measured by the Garmin Vivosmart 5 before the intervention, after the intervention, and at the 6-month follow-up, using an established protocol [71,92,96]. We exclude nonadherent or invalid days of wear as well as the highest and lowest values to reduce the influence of outliers. A minimum of 3 valid days of wear at each time point is required to calculate the averages. Sleep metrics include total hours asleep and sleep efficiency (the ratio of time asleep to time spent in bed) using guidelines for older adults [185]. We visually inspect sleep-wake patterns during the day to exclude naps from the sleep calculations [186].

#### Exploratory Assessments

##### Multiday Boston Remote Assessment for Neurocognitive Health

We are exploring the feasibility of integrating daily mobile cognitive functioning assessments via the Boston Remote Assessment for Neurocognitive Health (BRANCH) platform [160,187,188]. The multiday paradigm using BRANCH can be self-administered without researcher supervision using any web-enabled device (mobile phone, tablet device, or computer). Similar to RBANS assessments, we provide an iPad for participants who do not own a web-enabled device to complete the multiday BRANCH tests. Participants complete 1 BRANCH test per day for 7 days during the 3 main assessment periods; they complete a unique version at each time point (before the intervention, after the intervention, and the 6-month follow-up). Participants complete a brief sequence of three visual associative memory tasks involving everyday objects: (1) Digit-Signs Test (correct number of street sign–number pairings identified), (2) Groceries Prices Test (correct number of grocery-price pairings identified), and (3) Face-Name Test (average of correct responses from 2 face-name pairings: first letter name recall and full name recall). Multiday learning curves to capture the speed and learning of BRANCH stimuli will be computed for each task [160]. Participants are instructed to take the multiday BRANCH tests in a quiet, distraction-free environment. Participants indicate a preferred time to complete the assessment once per day. They receive automated and secure SMS text messages or email reminders with a link to access the test. The administration of the multiday BRANCH tests takes approximately 12 minutes per day. The multiday BRANCH tests have demonstrated strong feasibility, reliability, validity, and sensitivity to subtle changes in learning and memory in preclinical AD/ADRD [160,189].

##### Daily Surveys

Participants receive daily surveys to indicate adherence and SCD symptoms during the intervention phase. Daily surveys are sent from Twilio (Twilio Inc), a messaging platform with REDCap integration. Participants can receive the surveys via telephone calls, SMS text messages, or secure email according to personal preference. Daily surveys for MHB participants include (1) logging mindfulness practice (*yes* or *no*), (2) program mindfulness skills practiced (all that apply), and (3) total time (in minutes) spent practicing these skills. Similar to other mindfulness RCTs [161], HEP participants are asked to report daily minutes spent journaling to control for the time and attention devoted to the active skills in MHB. Participants in both groups will provide a global rating of perceived SCD (*What is your level of cognitive functioning right now?*) on an 11-point Likert scale ranging from 0=*bad: my thinking is very difficult or slow* to 10=*good: my thinking is sharp and quick* [162,163]. Participants may opt out of messages for the daily surveys and switch to a paper-and-pencil log.

##### Randomization

One week before the first session of MHB or the HEP, the RA randomizes participants to receive either 8 weeks of the MHB intervention or the HEP education control. Randomization follows a 1:1 ratio using permuted blocks of size 4. To maintain blinding of group assignments, participants are informed that they can participate in 1 of 2 programs: MHB 1 (active intervention [MHB]) or MHB 2 (control [HEP]). To reduce contamination, we ask participants not to share specific information discussed in the group (eg, skills learned and topics discussed) with anyone for the duration of the study. All participants will receive both electronic and paper copies of their assigned treatment manual.

### Treatment Arms

#### MHB Intervention

**Figure 3:**
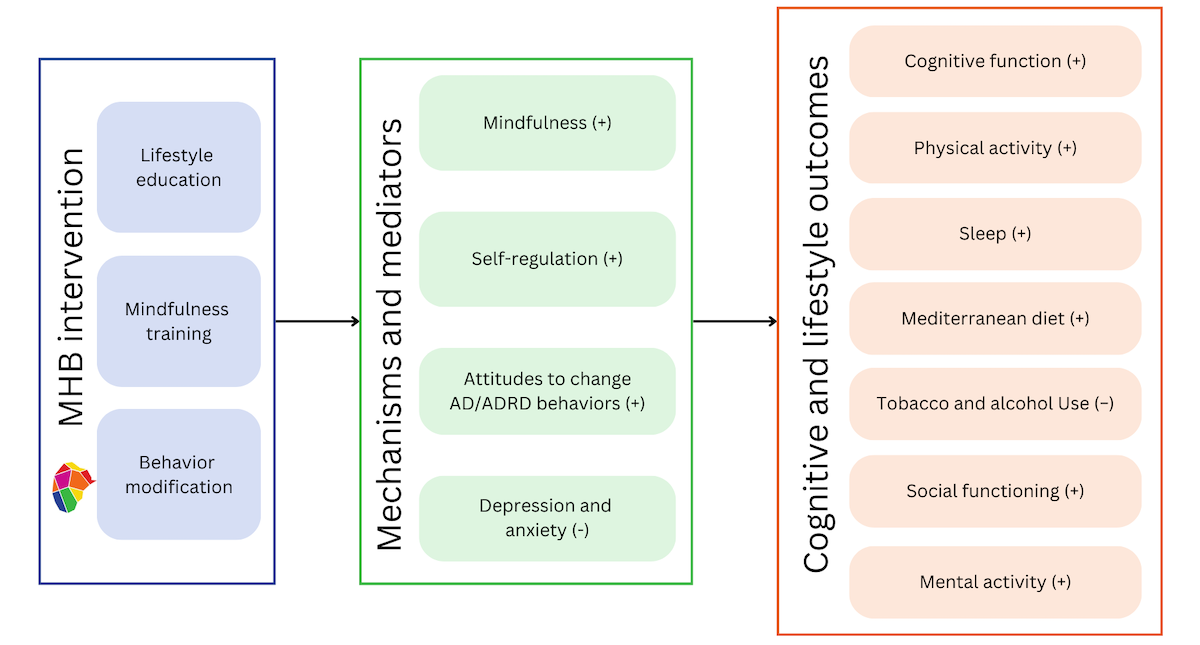
Conceptual model of My Healthy Brain (MHB). AD/ADRD: Alzheimer disease and Alzheimer disease–related dementias.

The iterative development of MHB has followed the NIH Stage Model [62] and Science of Behavior Change [190] frameworks. The objective of MHB is to increase the uptake and maintenance of lifestyle behavior modification to reduce the risk of AD/ADRD and cognitive decline among older adults, fostering long-term brain health and well-being (Figure 3). This objective aligns with the Scaffolding Theory of Aging and Cognition [191], which posits that lifestyle behaviors enhance “neural compensatory scaffolding,” thereby preserving brain structure, function, and cognition with aging. MHB targets *modifiable* lifestyle behaviors identified in our meta-analysis [63], relevant literature on modifiable AD/ADRD risk factors [22,192,193], and similar multidomain lifestyle interventions [30,31]. Modifiable lifestyle behaviors include physical activity, adherence to a Mediterranean diet, sleep quality, alcohol use, tobacco cessation, social functioning, and mental activity. MHB does not target other AD/ADRD risk factors that are not modifiable through a group-based behavioral intervention (eg, a history of head injury or exposure to air pollution [22]).

The intervention design and procedures were informed by MHB preliminary studies [64-66] and a similar virtual program for older adults with early cognitive decline [74,81,83,92,194]. MHB is delivered via 90-minute Zoom meetings in small groups of 5 to 10 older adults with early AD/ADRD risk (refer to Textbox 1 for the eligibility criteria). The MHB program is led by a clinical health psychologist with aging expertise and a supervised clinical psychology doctoral trainee. All sessions provide education on AD/ADRD risk factors and teach evidence-based behavior modification skills grounded in cognitive behavioral therapy [195] and mindful self-regulation theory [38-40]. Guided by qualitative work with older adults [71,96], we adapted all program skills to account for challenges related to SCD symptoms and aging (eg, forgetfulness, mobility issues, and loneliness). Textbox 2 contains a full list of topics for each MHB session. Participants receive a copy of the manual along with access to the program website, which includes educational videos, recordings of mindfulness skills, and additional resources.

In the first session, the group leader provides an overview of the program, sets expectations for participation (eg, attendance, home practice, and appropriate group behavior), defines key terms (eg, dementia, brain health, and mindfulness), and allows group members to introduce themselves. The remainder of the first session and all subsequent sessions follow the same agenda: review previous material (10% of session time), problem-solve adherence barriers (10%), discuss progress toward participants’ goals (10%), provide education on AD/ADRD risk factors (30%), and practice and apply behavior modification and mindfulness skills (40%). Group members are encouraged to collaboratively support each other by sharing strategies for coping with SCD symptoms and achieving lifestyle goals. MHB group participants wear the Garmin Vivosmart 5 to increase their daily step count gradually and safely following a quota-based pacing protocol (10% increase each week if step goal is achieved) [83]. At the end of each session, participants set an individualized specific, measurable, achievable, relevant, and timely (SMART) lifestyle behavioral goal [196]. During the week between MHB sessions, participants execute their SMART goal and practice mindfulness (5-10 min/d).

**Session outline for My Healthy Brain (MHB) and the health enhancement program (HEP).**

**MHB sessions and content**

- Session 1: Brain health and mindfulness
  Education: define brain health, bust common myths about brain health, identify protective lifestyle factors, and understand the benefits of mindfulness
  Mindfulness: practice “mindful awareness of breath” meditation
  Behavioral: reflect on motivations for participating, assess current brain health habits, and set lifestyle goals for the program
- Session 2: Physical activity and walking
  Education: understand the importance of physical activity (walking) for brain health, and use a wearable device (Garmin Vivosmart 5 watch) to monitor and reinforce my daily step count
  Mindfulness: pay attention nonjudgmentally by practicing “body scan” meditation
  Behavioral: set realistic and achievable walking goals (quota-based pacing), and link activities to enjoyment and purpose
- Session 3: Quality sleep
  Education: understand the importance of sleep for brain health, and use a wearable device (Garmin Vivosmart 5 watch) to monitor sleep (total time and efficiency)
  Mindfulness: learn how mindful responding can lead to healthier lifestyle choices through the “mindful STOP (stop, take a breath, observe, and proceed)” meditation
  Behavioral: use behavioral sleep hygiene strategies and problem-solve barriers to getting quality sleep
- Session 4: Mediterranean and Mediterranean–Dietary Approaches to Stop Hypertension Intervention for Neurodegenerative Delay (MIND) diet
  Education: understand the importance of the Mediterranean or MIND diet for brain health
  Mindfulness: bring mindfulness to my daily experiences through “mindful eating” meditation
  Behavioral: notice hunger and fullness urges to reduce overeating, and track dietary changes using a MIND diet log
- Session 5: Mental activity and cognitive reserve
  Education: understand the importance of being mentally active for brain health, and identify cognitive strengths and weaknesses
  Mindfulness: learn the brain health benefits of mindfulness and practice “bringing awareness to unwanted experiences” meditation
  Behavioral: develop compensatory strategies for memory-related problems (MRPs), and get mentally active to build cognitive reserve
- Session 6: Social activity and brain health
  Education: understand the importance of social activities for brain health and the risks of loneliness
  Mindfulness: practice mindful communication to improve my relationships, openness, and compassion to myself and others; and practice “love and kindness” meditation
  Behavioral: brainstorm ways to become more socially active, and create shared activity plans to reduce loneliness
- Session 7: Mindfulness of unhealthy urges
  Education: understand the impacts of alcohol, tobacco, and substance use on brain health, identify urges that become unhealthy habits
  Mindfulness: practice tolerating urges with the “urge surfing” meditation
  Behavioral: break unhealthy habits by identifying environments and stressors that derail lifestyle goals
- Session 8: Maintaining a brain-healthy life
  Education: understand how to sustain a healthy lifestyle and prepare for the end of MHB
  Mindfulness: review how to integrate mindfulness into daily life and practice “mountain” meditation
  Behavioral: evaluate progress in the program and develop a plan to main acquired skills

**HEP sessions and content**

- Session 1: Program overview and MRPs
  Program goals, understand MRPs, how stress and MRPs are connected, and the impact of stress
- Session 2: The connection between MRPs and physical wellness
  Understand the connection between MRPs and physical wellness
- Session 3: Sleep and wellness—connection with MRPs
  Healthy sleeping strategies and cognitive and physical health
- Session 4: Exercise and wellness—connection with MRPs
  Physical exercise, maintaining healthy weight, and tips for getting active
- Session 5: Nutrition—connection with MRPs
  Basic nutrition, unique needs and tips for older adults, and portion size
- Session 6: Substance use, supplements, and medications—connection with MRPs
  The impact of tobacco and alcohol use on brain health and aging, guidance on over-the-counter memory supplements, and medication management
- Session 7: Social support and loneliness—connection with MRPs
  The impact of social isolation on brain health, types of social support, and physician support
- Session 8: Program review
  Overview of program content

#### HEP Control

The HEP is a time- and attention-matched education control [68,69]. The HEP has been used in similar RCTs, including those involving older adults with SCD [74,81,83,92,197-201]. This active control accounts for the effect of time spent as well as feedback and support from group members and the study clinician. Participants receive lifestyle education consistent with public health recommendations and standards for health promotion (eg, physical activity, sleep, and the Mediterranean diet; Textbox 2). Participants do not learn the mindfulness or behavior modification skills unique to MHB. To control for the time and attention devoted to MHB skills, HEP participants are instructed to journal for 5 to 10 minutes per day. HEP participants wear the Garmin Vivosmart 5 but do not monitor steps and sleep or set goals (ie, these features are disabled in the Garmin Connect app).

### Data Analyses

#### Power Analysis

Consistent with the NIH Stage Model [62] and guidelines for feasibility pilot studies [76,77], the pilot RCT trial is primarily focused on feasibility benchmarks and *not* efficacy testing. With a sample size of 50 participants and assuming conservatively that the 10 feasibility criteria are independent, the study will have >80% power to confirm all benchmarks if at least 82% of the participants meet the specified benchmark for each criterion. The proposed sample size is consistent with prior similar pilot trials [83].

#### Primary Analyses (Feasibility)

Statistical analyses will be performed in R (version 4.2.1; R Foundation for Statistical Computing) using RStudio (Posit Software, PBC) [202,203]. We will calculate frequencies and proportions to assess a priori feasibility Go–No-Go benchmarks for the feasibility, acceptability, appropriateness, credibility, satisfaction, and fidelity of the programs [64,70-74]. If these benchmarks are not met, revisions will be necessary before an efficacy trial. We will report benchmarks separately for the MHB intervention and HEP control groups.

#### Exploratory Analyses

Consistent with the Science of Behavior Change framework [189], we will test preliminary improvements and explore mechanisms to guide intervention refinements before efficacy testing. For each measure, we will report descriptive statistics, paired samples 2-tailed *t* tests, and Cohen *d* effect sizes (small=0.2, medium=0.5, and large=0.8) [204] with 95% CIs and minimal clinically important difference where available. We will analyze the MHB and HEP groups separately. We will explore associations between hypothesized mechanisms and outcomes to gauge the evidence of engagement in the targets of the MHB intervention. Finally, we will examine adherence and person-specific patterns of intensive longitudinal data (Garmin Vivosmart 5, multiday BRANCH tests, and daily surveys) during the RCT.

## Results

### Overview

This study was funded by a National Institute on Aging Mentored Patient-Oriented Research Career Development Award (K23; 1 K23 AG075257-01; September 2022). The study was approved by the IRB in October 2023. We began recruitment in January 2024. As of June 1, 2024, a total of 225 individuals had inquired about the study via self-referral or referral from a clinician or community partner. We made initial contact and conducted screenings with 213 (94.7%) of the 225 individuals; 25.6% (40/156) met the eligibility criteria. Of the 40 eligible participants, 21 (52.5%) were enrolled in 2 group cohorts, 17 (42.5%) were on hold for future group cohorts, and 2 (5%) withdrew before enrollment. Primary reasons for ineligibility included low AD/ADRD risk (CAIDE score <6; 54/116, 46.6%), active lifestyle (>5000 average steps per day and ≥30 minutes of exercise per day; 26/116, 22.4%), no self-reported SCD (18/116, 15.5%), age <60 years (16/116, 13.8%), regular mindfulness practice (8/116, 6.9%), and clinically significant cognitive impairment (Telephone Interview for Cognitive Status score <31 out of a maximum possible 41 points; 5/116, 4.3%).

All 21 enrolled participants were randomized to MHB (n=11, 52%) or the HEP (n=10, 48%) across 2 group cohorts (cohort 1: n=9, 43%; cohort 2: n=12, 57%). All participants completed before the intervention assessments. No participants have dropped out after enrollment. All participants in our first cohort completed ≥6 out of 8 sessions of either MHB or the HEP (including minimal makeup sessions) and after the intervention assessments (9/9, 100%). One participant requested to skip the HEP session on substance use due to a traumatic family history. The intervention for our second cohort (12/21, 57%) is ongoing (5 out of 8 sessions completed; 100% attendance with minimal makeup sessions). Across both cohorts, adherence rates are high for the Garmin Vivosmart 5 (128/147, 87.1% total weeks) and daily surveys (105/122, 86.1% total weeks). We plan to complete enrollment by December 2024 and data analyses by December 2025.

### Demographics

Sample characteristics for enrolled cohorts 1 and 2 are presented in Table 3.

**Table 3:** Demographics of enrolled and scheduled My Healthy Brain study participants (n=21).

Characteristics		Values
Age (y), mean (SD; range)		72 (7.71; 60-88)
**Sex, n (%)**		
	Female	16 (76)
	Male	5 (24)
	Chose not to answer	0 (0)
**Gender, n (%)**		
	Women	16 (76)
	Men	5 (24)
	Nonbinary	0 (0)
	Chose not to answer	0 (0)
**Ethnicity, n (%)**		
	Hispanic or Latinx	1 (5)
	Not Hispanic or Latinx	19 (91)
	Chose not to answer	1 (5)
**Race, n (%)**		
	Asian or Asian American	1 (5)
	Black or African American	1 (5)
	White	17 (81)
	Multiracial	0 (0)
	Chose not to answer	2 (10)
**Marital status, n (%)**		
	Single, never married	2 (11)
	Married	9 (43)
	Living with significant other	0 (0)
	Separated or divorced	5 (24)
	Widowed	5 (24)
	Chose not to answer	0 (0)
**Living status, n (%)**		
	Live alone	7 (33)
	Live alone with spouse or partner	9 (43)
	Live alone with 1 other friend or roommate	2 (10)
	Live with caregiver	0 (0)
	Live with group (private residence)	2 (10)
	Live in a group home	1 (5)
	Other	0 (0)
	Chose not to answer	0 (0)
**Education, n (%)**		
	Completed high school or GED^a^ (12 y)	1 (5)
	Some college or associate’s degree (<16 y)	2 (10)
	Completed 4 years of college (16 y)	8 (38)
	Graduate or professional degree (>16 y)	10 (48)
	Chose not to answer	0 (0)
**Employment status, n (%)**		
	Employed full time	5 (24)
	Employed part time	4 (19)
	Retired	12 (57)
	Other	0 (0)
	Chose not to answer	0 (0)
**Income (US $), n (%)**		
	<10,000	1 (5)
	10,000 to <15,000	0 (0)
	15,000 to <20,000	0 (0)
	20,000 to <25,000	3 (14)
	25,000 to <35,000	1 (5)
	35,000 to <50,000	3 (14)
	50,000 to <75,000	6 (29)
	75,000 to <100,000	3 (14)
	≥100,000	4 (19)
	Chose not to answer	0 (0)

^a^GED: General Educational Development.

## Discussion

### Summary

SCD represents a critical opportunity for early intervention to modify risk factors for AD/ADRD. MHB addresses this gap by offering a novel group mindfulness-based lifestyle program tailored to older adults with SCD. Single-arm open pilot studies (NIH stage 1A) have demonstrated the feasibility and acceptability of MHB, indicating its potential to bypass challenges to lifestyle behavior modification and improve cognitive outcomes among older adults. These preliminary studies also revealed barriers to participation that must be addressed during the design and execution of the first RCT of MHB before conducting fully powered trials or implementing the program in clinical care.

The primary aim of this feasibility RCT is to establish the feasibility, acceptability, appropriateness, credibility, satisfaction, and fidelity of the MHB intervention compared to the HEP active control (NIH stage 1B). Our secondary aim is to explore preliminary signals of improvement in outcomes and hypothesized mechanisms (Table 1 and Figure 3). Testing these aims and our ability to randomize participants to MHB versus the HEP will serve as a “dress rehearsal” for a subsequent efficacy RCT. In addition, this RCT will provide valuable insights into the feasibility of integrating multiple digital health technologies into a lifestyle trial for older adults with SCD. If feasible, the novel integration of passive activity sensors, repeated mobile cognitive assessments, and daily surveys offer a comprehensive framework for monitoring adherence and understanding individual responses to lifestyle interventions and the mechanisms of action. This is notable because the pathways by which lifestyle interventions may improve cognitive outcomes and prevent AD/ADRD among older adults with SCD are poorly understood.

We have made several changes to our study protocol (Multimedia Appendix 1) guided by our qualitative studies [67]. Using implementation science frameworks [205-207] and the socioecological model [208], the protocol changes and strategies aim to maximize RCT outcomes, increase sample diversity within our RCT, and reduce barriers to AD/ADRD prevention efforts [209-212]. Initial observations suggest that our protocol changes and strategies have been effective. We have observed a high volume of inquiries, indicating that our outreach efforts have been successful and that older adults are interested in participating in the study. Participant adherence to both programs is notably high, with positive engagement in the mindfulness and behavior change skills taught in MHB. Preliminary Garmin Vivosmart 5 and daily survey adherence data are also promising but must be confirmed upon study completion. Providing individualized support and devices at no cost has enabled us to include and retain older adults with limited technological access or proficiency [209,210,213]. To date, the enrolled participants predominantly represent White, well-educated women. In our next group cohorts, we will prioritize the inclusion of racial and ethnic minoritized older adults to address their underrepresentation in prevention RCTs and their increased risk of AD/ADRD [101,102]. We are conducting broader outreach, including both professional and community groups, to further increase awareness and diverse participation [214,215]. The feasibility RCT will provide initial information on our ability to engage older adult communities in AD/ADRD prevention research.

### Limitations

Despite the protocol improvements, several limitations remain and are outlined in the following subsections (additional limitations may arise during the execution of our trial).

#### Sample Diversity

While we have increased the diversity of our recruitment sources through community outreach, it is difficult to enroll racial and ethnic minoritized older adults. It takes time to build trust in community partnerships and address barriers to participation in clinical trials rooted in social determinants of health [216]. In addition, our interventions are currently only available in English, which greatly limits our ability to include many older adults in our community who speak Spanish or other languages. To address disparities in AD/ADRD prevention, future studies will incorporate principles of community-based participatory research and set benchmarks for enrolling minoritized older adults.

#### Digital Health Divide

We have designed a more digitally inclusive virtual RCT by providing devices and individualized technological support. However, the RCT fails to serve older adults who are disinterested in virtual interventions, lack broadband internet access, or experience other challenges with technology (eg, visual or hearing impairments).

#### Identification of Early AD/ADRD Risk

We aim to identify older adults with the earliest preclinical AD/ADRD, determined by the presence of cardiovascular risk factors (CAIDE scores) and rigorous criteria for SCD [8], similar to other lifestyle trials [30,31,35]. However, SCD presents as unspecific symptoms and can be attributed to normal cognitive aging, psychiatric disorders (eg, depression and anxiety), sleep disturbances, and other conditions rather than neurodegeneration [8]. The development of meaningful cognitive markers is increasingly important in interventions aiming to modify early risk of AD/ADRD, such as MHB.

### Conclusions

The MHB feasibility RCT represents a significant step toward developing practical, evidence-based interventions for early AD/ADRD prevention. By addressing key benchmarks and engagement in intervention targets, this study lays the groundwork for larger trials aimed at validating the potential long-term benefits of mindfulness-based lifestyle programs in reducing dementia risk. Following the NIH Stage Model, the next phase of intervention development will evaluate MHB versus the HEP in a larger RCT and more diverse sample (NIH stage 2). We will rigorously test the superiority of MHB versus the HEP in enhancing cognitive and lifestyle outcomes among older adults with SCD and modifiable AD/ADRD risk factors. If successful, MHB could provide an effective and scalable intervention for reducing AD/ADRD risk, leveraging digital health technology to promote sustained behavior change and brain health.

## Appendix

Multimedia Appendix 1 Summary of protocol changes and strategies to enhance randomized controlled trial outcomes.

## Abbreviations

AD/ADRD: Alzheimer disease and Alzheimer disease–related dementias
BRANCH: Boston Remote Assessment for Neurocognitive Health
CAIDE: Cardiovascular Risk Factors, Aging, and Incidence of Dementia
HEP: health enhancement program
IRB: institutional review board
MGB: Mass General Brigham
MHB: My Healthy Brain
NIH: National Institutes of Health
RA: research assistant
RBANS: Repeatable Battery for the Assessment of Neuropsychological Status
RCT: randomized controlled trial
REDCap: Research Electronic Data Capture
SCD: subjective cognitive decline
SMART: specific, measurable, achievable, relevant, and timely

## Data Availability

The datasets generated and analyzed during this study are available from the corresponding author on reasonable request.

## References

[1] Grand JH, Caspar S, Macdonald SW (2011). Clinical features and multidisciplinary approaches to dementia care. J Multidiscip Healthc.

[2] Cattaneo G, Bartrés-Faz D, Morris TP, Sánchez JS, Macià D, Tarrero C, Tormos JM, Pascual-Leone A (2018). The Barcelona Brain Health Initiative: a cohort study to define and promote determinants of brain health. Front Aging Neurosci.

[3] Gorelick PB, Furie KL, Iadecola C, Smith EE, Waddy SP, Lloyd-Jones DM, Bae HJ, Bauman MA, Dichgans M, Duncan PW, Girgus M, Howard VJ, Lazar RM, Seshadri S, Testai FD, van Gaal S, Yaffe K, Wasiak H, Zerna C, American Heart Association/American Stroke Association (2017). Defining optimal brain health in adults: a presidential advisory from the American Heart Association/American Stroke Association. Stroke.

[4] Gelfo F, Mandolesi L, Serra L, Sorrentino G, Caltagirone C (2018). The neuroprotective effects of experience on cognitive functions: evidence from animal studies on the neurobiological bases of brain reserve. Neuroscience.

[5] Alzheimer's disease facts and figures. Alzheimer's Association.

[6] Stewart R (2012). Subjective cognitive impairment. Curr Opin Psychiatry.

[7] Fonseca JA, Ducksbury R, Rodda J, Whitfield T, Nagaraj C, Suresh K, Stevens T, Walker Z (2015). Factors that predict cognitive decline in patients with subjective cognitive impairment. Int Psychogeriatr.

[8] Jessen F, Amariglio RE, van Boxtel M, Breteler M, Ceccaldi M, Chételat G, Dubois B, Dufouil C, Ellis KA, van der Flier WM, Glodzik L, van Harten AC, de Leon MJ, McHugh P, Mielke MM, Molinuevo JL, Mosconi L, Osorio RS, Perrotin A, Petersen RC, Rabin LA, Rami L, Reisberg B, Rentz DM, Sachdev PS, de la Sayette V, Saykin AJ, Scheltens P, Shulman MB, Slavin MJ, Sperling RA, Stewart R, Uspenskaya O, Vellas B, Visser PJ, Wagner M, Subjective Cognitive Decline Initiative (SCD-I) Working Group (2014). A conceptual framework for research on subjective cognitive decline in preclinical Alzheimer's disease. Alzheimers Dement.

[9] Molinuevo JL, Rabin LA, Amariglio R, Buckley R, Dubois B, Ellis KA, Ewers M, Hampel H, Klöppel S, Rami L, Reisberg B, Saykin AJ, Sikkes S, Smart CM, Snitz BE, Sperling R, van der Flier WM, Wagner M, Jessen F, Subjective Cognitive Decline Initiative (SCD-I) Working Group (2016). Implementation of subjective cognitive decline criteria in research studies. Alzheimers Dement.

[10] Jessen F (2014). Subjective and objective cognitive decline at the pre-dementia stage of Alzheimer's disease. Eur Arch Psychiatry Clin Neurosci.

[11] Verlinden VJ, van der Geest JN, de Bruijn RF, Hofman A, Koudstaal PJ, Ikram MA (2016). Trajectories of decline in cognition and daily functioning in preclinical dementia. Alzheimers Dement.

[12] Studart A, Nitrini R (2016). Subjective cognitive decline: the first clinical manifestation of Alzheimer's disease?. Dement Neuropsychol.

[13] Mitchell AJ, Beaumont H, Ferguson D, Yadegarfar M, Stubbs B (2014). Risk of dementia and mild cognitive impairment in older people with subjective memory complaints: meta-analysis. Acta Psychiatr Scand.

[14] Andel R, Crowe M, Pedersen NL, Fratiglioni L, Johansson B, Gatz M (2008). Physical exercise at midlife and risk of dementia three decades later: a population-based study of Swedish twins. J Gerontol A Biol Sci Med Sci.

[15] Baumgart M, Snyder HM, Carrillo MC, Fazio S, Kim H, Johns H (2015). Summary of the evidence on modifiable risk factors for cognitive decline and dementia: a population-based perspective. Alzheimers Dement.

[16] Geda YE, Roberts RO, Knopman DS, Christianson TJ, Pankratz VS, Ivnik RJ, Boeve BF, Tangalos EG, Petersen RC, Rocca WA (2010). Physical exercise, aging, and mild cognitive impairment: a population-based study. Arch Neurol.

[17] Hamer M, Chida Y (2009). Physical activity and risk of neurodegenerative disease: a systematic review of prospective evidence. Psychol Med.

[18] Xu W, Tan L, Wang HF, Jiang T, Tan MS, Tan L, Zhao QF, Li JQ, Wang J, Yu JT (2015). Meta-analysis of modifiable risk factors for Alzheimer's disease. J Neurol Neurosurg Psychiatry.

[19] Barnes DE, Yaffe K (2011). The projected effect of risk factor reduction on Alzheimer's disease prevalence. Lancet Neurol.

[20] Cooper C, Li R, Lyketsos C, Livingston G (2013). Treatment for mild cognitive impairment: systematic review. Br J Psychiatry.

[21] (2023). The epidemiology and impact of dementia: current state and future trends. World Health Organization.

[22] Livingston G, Huntley J, Sommerlad A, Ames D, Ballard C, Banerjee S, Brayne C, Burns A, Cohen-Mansfield J, Cooper C, Costafreda SG, Dias A, Fox N, Gitlin LN, Howard R, Kales HC, Kivimäki M, Larson EB, Ogunniyi A, Orgeta V, Ritchie K, Rockwood K, Sampson EL, Samus Q, Schneider LS, Selbæk G, Teri L, Mukadam N (2020). Dementia prevention, intervention, and care: 2020 report of the Lancet Commission. Lancet.

[23] Jones A, Ali MU, Kenny M, Mayhew A, Mokashi V, He H, Lin S, Yavari E, Paik K, Subramanian D, Dydynsky R, Aryal K, Correia RH, Dash D, Manis DR, O'Connell M, Liu-Ambrose T, Taler V, McMillan JM, Hogan DB, Kirkland S, Costa AP, Wolfson C, Raina P, Griffith L (2024). Potentially modifiable risk factors for dementia and mild cognitive impairment: an umbrella review and meta-analysis. Dement Geriatr Cogn Disord.

[24] Shi L, Chen SJ, Ma MY, Bao YP, Han Y, Wang YM, Shi J, Vitiello MV, Lu L (2018). Sleep disturbances increase the risk of dementia: a systematic review and meta-analysis. Sleep Med Rev.

[25] Wang S, Zheng X, Huang J, Liu J, Li C, Shang H (2024). Sleep characteristics and risk of Alzheimer's disease: a systematic review and meta-analysis of longitudinal studies. J Neurol.

[26] Loughrey DG, Lavecchia S, Brennan S, Lawlor BA, Kelly ME (2017). The impact of the mediterranean diet on the cognitive functioning of healthy older adults: a systematic review and meta-analysis. Adv Nutr.

[27] Su S, Shi L, Zheng Y, Sun Y, Huang X, Zhang A, Que J, Sun X, Shi J, Bao Y, Deng J, Lu L (2022). Leisure activities and the risk of dementia: a systematic review and meta-analysis. Neurology.

[28] Yates LA, Ziser S, Spector A, Orrell M (2016). Cognitive leisure activities and future risk of cognitive impairment and dementia: systematic review and meta-analysis. Int Psychogeriatr.

[29] Radd-Vagenas S, Duffy SL, Naismith SL, Brew BJ, Flood VM, Fiatarone Singh MA (2018). Effect of the Mediterranean diet on cognition and brain morphology and function: a systematic review of randomized controlled trials. Am J Clin Nutr.

[30] Ngandu T, Lehtisalo J, Solomon A, Levälahti E, Ahtiluoto S, Antikainen R, Bäckman L, Hänninen T, Jula A, Laatikainen T, Lindström J, Mangialasche F, Paajanen T, Pajala S, Peltonen M, Rauramaa R, Stigsdotter-Neely A, Strandberg T, Tuomilehto J, Soininen H, Kivipelto M (2015). A 2 year multidomain intervention of diet, exercise, cognitive training, and vascular risk monitoring versus control to prevent cognitive decline in at-risk elderly people (FINGER): a randomised controlled trial. Lancet.

[31] McMaster M, Kim S, Clare L, Torres SJ, Cherbuin N, DʼEste C, Anstey KJ (2020). Lifestyle risk factors and cognitive outcomes from the multidomain dementia risk reduction randomized controlled trial, body brain life for cognitive decline (BBL-CD). J Am Geriatr Soc.

[32] Moll van Charante EP, Richard E, Eurelings LS, van Dalen JW, Ligthart SA, van Bussel EF, Hoevenaar-Blom MP, Vermeulen M, van Gool WA (2016). Effectiveness of a 6-year multidomain vascular care intervention to prevent dementia (preDIVA): a cluster-randomised controlled trial. Lancet.

[33] Lam LC, Chan WC, Leung T, Fung AW, Leung EM (2015). Would older adults with mild cognitive impairment adhere to and benefit from a structured lifestyle activity intervention to enhance cognition?: a cluster randomized controlled trial. PLoS One.

[34] Andrieu S, Guyonnet S, Coley N, Cantet C, Bonnefoy M, Bordes S, Bories L, Cufi MN, Dantoine T, Dartigues JF, Desclaux F, Gabelle A, Gasnier Y, Pesce A, Sudres K, Touchon J, Robert P, Rouaud O, Legrand P, Payoux P, Caubere JP, Weiner M, Carrié I, Ousset PJ, Vellas B, MAPT Study Group (2017). Effect of long-term omega 3 polyunsaturated fatty acid supplementation with or without multidomain intervention on cognitive function in elderly adults with memory complaints (MAPT): a randomised, placebo-controlled trial. Lancet Neurol.

[35] Cooper C, Aguirre E, Barber JA, Bass N, Brodaty H, Burton A, Higgs P, Hunter R, Huntley J, Lang I, Kales HC, Marchant NL, Minihane AM, Ritchie K, Morgan-Trimmer S, Walker Z, Walters K, Wenborn J, Rapaport P (2020). APPLE-tree (active prevention in people at risk of dementia: lifestyle, bEhaviour change and technology to REducE cognitive and functional decline) programme: protocol. Int J Geriatr Psychiatry.

[36] Coley N, Ngandu T, Lehtisalo J, Soininen H, Vellas B, Richard E, Kivipelto M, Andrieu S, HATICE‚ FINGER‚MAPT/DSA groups (2019). Adherence to multidomain interventions for dementia prevention: data from the FINGER and MAPT trials. Alzheimers Dement.

[37] Ludwig DS, Kabat-Zinn J (2008). Mindfulness in medicine. JAMA.

[38] Geiger PJ, Boggero IA, Brake CA, Caldera CA, Combs HL, Peters JR, Baer RA (2016). Mindfulness-based interventions for older adults: a review of the effects on physical and emotional well-being. Mindfulness (N Y).

[39] Dutton G (2008). The role of mindfulness in health behavior change. Acsms Health Fit J.

[40] Victorson D, Kentor M, Maletich C, Lawton RC, Kaufman VH, Borrero M, Languido L, Lewett K, Pancoe H, Berkowitz C (2014). Mindfulness meditation to promote wellness and manage chronic disease: a systematic review and meta-analysis of mindfulness-based randomized controlled trials relevant to lifestyle medicine. Am J Lifestyle Med.

[41] Hölzel BK, Carmody J, Vangel M, Congleton C, Yerramsetti SM, Gard T, Lazar SW (2011). Mindfulness practice leads to increases in regional brain gray matter density. Psychiatry Res.

[42] Sevinc G, Hölzel BK, Greenberg J, Gard T, Brunsch V, Hashmi JA, Vangel M, Orr SP, Milad MR, Lazar SW (2019). Strengthened hippocampal circuits underlie enhanced retrieval of extinguished fear memories following mindfulness training. Biol Psychiatry.

[43] Gotink RA, Meijboom R, Vernooij MW, Smits M, Hunink MG (2016). 8-week mindfulness based stress reduction induces brain changes similar to traditional long-term meditation practice - a systematic review. Brain Cogn.

[44] Tang YY, Hölzel BK, Posner MI (2015). The neuroscience of mindfulness meditation. Nat Rev Neurosci.

[45] Creswell JD, Lindsay EK (2014). How does mindfulness training affect health? A mindfulness stress buffering account. Curr Dir Psychol Sci.

[46] Kilpatrick LA, Suyenobu BY, Smith SR, Bueller JA, Goodman T, Creswell JD, Tillisch K, Mayer EA, Naliboff BD (2011). Impact of mindfulness-based stress reduction training on intrinsic brain connectivity. Neuroimage.

[47] Gard T, Hölzel BK, Lazar SW (2014). The potential effects of meditation on age-related cognitive decline: a systematic review. Ann N Y Acad Sci.

[48] Prakash RS (2021). Mindfulness meditation: impact on attentional control and emotion dysregulation. Arch Clin Neuropsychol.

[49] Smart CM, Segalowitz SJ, Mulligan BP, Koudys J, Gawryluk JR (2016). Mindfulness training for older adults with subjective cognitive decline: results from a pilot randomized controlled trial. J Alzheimers Dis.

[50] Russell-Williams J, Jaroudi W, Perich T, Hoscheidt S, El Haj M, Moustafa AA (2018). Mindfulness and meditation: treating cognitive impairment and reducing stress in dementia. Rev Neurosci.

[51] Wetherell JL, Hershey T, Hickman S, Tate SR, Dixon D, Bower ES, Lenze EJ (2017). Mindfulness-based stress reduction for older adults with stress disorders and neurocognitive difficulties: a randomized controlled trial. J Clin Psychiatry.

[52] Lenze EJ, Hickman S, Hershey T, Wendleton L, Ly K, Dixon D, Doré P, Wetherell JL (2014). Mindfulness-based stress reduction for older adults with worry symptoms and co-occurring cognitive dysfunction. Int J Geriatr Psychiatry.

[53] Herring A, Blome M, AmbrÃ©e O, Sachser N, Paulus W, Keyvani K (2010). Reduction of cerebral oxidative stress following environmental enrichment in mice with Alzheimer-like pathology. Brain Pathol.

[54] Bremner JD (1999). Does stress damage the brain?. Biol Psychiatry.

[55] Deshmukh VD, Deshmukh SV (1990). Stress-adaptation failure hypothesis of Alzheimer's disease. Med Hypotheses.

[56] Innes KE, Selfe TK (2014). Meditation as a therapeutic intervention for adults at risk for Alzheimer's disease - potential benefits and underlying mechanisms. Front Psychiatry.

[57] de Frias CM, Whyne E (2015). Stress on health-related quality of life in older adults: the protective nature of mindfulness. Aging Ment Health.

[58] Parra DC, Wetherell JL, Van Zandt A, Brownson RC, Abhishek J, Lenze EJ (2019). A qualitative study of older adults' perspectives on initiating exercise and mindfulness practice. BMC Geriatr.

[59] Zeidan F, Johnson SK, Diamond BJ, David Z, Goolkasian P (2010). Mindfulness meditation improves cognition: evidence of brief mental training. Conscious Cogn.

[60] Malinowski P, Moore AW, Mead BR, Gruber T (2017). The effects of regular brief mindfulness practice on electrophysiological markers of cognitive and affective processing in older adults. Mindfulness (N Y).

[61] Polsinelli AJ, Kaszniak AW, Glisky EL, Ashish D (2020). Effects of a brief, online, focused attention mindfulness training on cognition in older adults: a randomized controlled trial. Mindfulness.

[62] Onken LS, Carroll KM, Shoham V, Cuthbert BN, Riddle M (2014). Reenvisioning clinical science: unifying the discipline to improve the public health. Clin Psychol Sci.

[63] Mace RA, Stauder MJ, Hopkins SW, Cohen JE, Pietrzykowski MO, Philpotts LL, Luberto CM, Vranceanu AM (2024). Mindfulness-based interventions targeting modifiable lifestyle behaviors associated with brain health: a systematic review and meta-analysis. Am J Lifestyle Med.

[64] Mace RA, Greenberg J, Stauder M, Reynolds G, Vranceanu AM (2022). My healthy brain: a multimodal lifestyle program to promote brain health. Aging Ment Health.

[65] Mace RA, Hopkins SW, Reynolds GO, Vranceanu AM (2022). My healthy brain: rationale and case report of a virtual group lifestyle program targeting modifiable risk factors for dementia. J Clin Psychol Med Settings.

[66] Mace RA, Popok PJ, Hopkins SW, Fishbein NS, Vranceanu AM (2022). Adaptation and virtual feasibility pilot of a mindfulness-based lifestyle program targeting modifiable dementia risk factors in older adults. Aging Ment Health.

[67] Mace RA, Lyons C, Cohen JE, Ritchie C, Bartels S, Okereke OI, Hoeppner BB, Brewer JA, Vranceanu AM (2024). Optimizing the implementation of a lifestyle dementia prevention intervention for older patients in an academic healthcare system. J Alzheimers Dis.

[68] Mahaffey BL, Mackin DM, Vranceanu AM, Lofaro L, Bromet EJ, Luft BJ, Gonzalez A (2018). The stony brook health enhancement program: the development of an active control condition for mind-body interventions. J Health Psychol.

[69] Vranceanu AM, Zale EL, Funes CJ, Macklin EA, McCurley J, Park ER, Jordan JT, Lin A, Plotkin SR (2018). Mind-body treatment for international English-speaking adults with neurofibromatosis via live videoconferencing: protocol for a single-blind randomized controlled trial. JMIR Res Protoc.

[70] Lester EG, Hopkins SW, Popok PJ, Vranceanu AM (2021). Adaptation of a live video mind-body program to a web-based platform for English-speaking adults with neurofibromatosis: protocol for the NF-web study. JMIR Res Protoc.

[71] Greenberg J, Lin A, Zale EL, Kulich RJ, James P, Millstein RA, Shapiro H, Schatman ME, Edwards RR, Vranceanu AM (2019). Development and early feasibility testing of a mind-body physical activity program for patients with heterogeneous chronic pain; the GetActive study. J Pain Res.

[72] Jacobs CA, Mace RA, Greenberg J, Popok PJ, Reichman M, Lattermann C, Burris JL, Macklin EA, Vranceanu AM (2021). Development of a mind body program for obese knee osteoarthritis patients with comorbid depression. Contemp Clin Trials Commun.

[73] Lester E, DiStefano S, Mace R, Macklin E, Plotkin S, Vranceanu AM (2020). Virtual mind-body treatment for geographically diverse youth with neurofibromatosis: a pilot randomized controlled trial. Gen Hosp Psychiatry.

[74] Mace RA, Doorley JD, Popok PJ, Vranceanu AM (2021). Live video adaptations to a mind-body activity program for chronic pain and cognitive decline: protocol for the virtual active brains study. JMIR Res Protoc.

[75] Kraemer HC (2003). "Rules" of evidence in assessing the efficacy and effectiveness of treatments. Dev Neuropsychol.

[76] Lancaster GA, Dodd S, Williamson PR (2004). Design and analysis of pilot studies: recommendations for good practice. J Eval Clin Pract.

[77] Leon AC, Davis LL, Kraemer HC (2011). The role and interpretation of pilot studies in clinical research. J Psychiatr Res.

[78] (2022). Resource guide: remote delivery of evidence-based programs. National Council on Aging.

[79] McDermott K, Bakhshaie J, Brewer J, Vranceanu AM (2024). The impact of a virtual mind-body program on symptoms of depression and anxiety among international English-speaking adults with neurofibromatosis. Am J Med Genet A.

[80] Wang KE, Vranceanu AM, Lester EG (2023). Resiliency outcomes after participation in an asynchronous web-based platform for adults with neurofibromatosis: the NF-web study. PLoS One.

[81] Choukas NR, Mace RA, Rochon EA, Brewer JR, Vranceanu AM (2024). Exploring mechanisms of improvement in the Active Brains intervention for older adults with chronic pain and early cognitive decline. Arch Gerontol Geriatr.

[82] Presciutti AM, Woodworth E, Rochon E, Neale M, Motta M, Piazza J, Vranceanu AM, Hwang DY (2023). A mindfulness-based resiliency program for caregivers of patients with severe acute brain injury transitioning out of critical care: protocol for an open pilot trial. JMIR Res Protoc.

[83] Vranceanu AM, Choukas NR, Rochon EA, Duarte B, Pietrzykowski MO, McDermott K, Hooker JE, Kulich R, Quiroz YT, Parker RA, Macklin EA, Ritchie C, Mace RA, Active Brains Project (2023). Addressing the chronic pain-early cognitive decline comorbidity among older adults: protocol for the active brains remote efficacy trial. JMIR Res Protoc.

[84] Woodworth EC, Briskin EA, Plys E, Macklin E, Tatar RG, Huberty J, Vranceanu AM (2023). Mindfulness-based app to reduce stress in caregivers of persons with Alzheimer disease and related dementias: protocol for a single-blind feasibility proof-of-concept randomized controlled trial. JMIR Res Protoc.

[85] Bannon S, Brewer J, Ahmad N, Cornelius T, Jackson J, Parker RA, Dams-O'Connor K, Dickerson BC, Ritchie C, Vranceanu AM (2023). A live video dyadic resiliency intervention to prevent chronic emotional distress early after dementia diagnoses: protocol for a dyadic mixed methods study. JMIR Res Protoc.

[86] Presciutti AM, Lester EG, Woodworth EC, Greenberg J, Bakhshaie J, Hooker JE, McDermott KA, Vranceanu AM (2023). The impact of a virtual mind-body program on resilience factors among international English-speaking adults with neurofibromatoses: secondary analysis of a randomized clinical trial. J Neurooncol.

[87] Vranceanu AM, Manglani HR, Choukas NR, Kanaya MR, Lester E, Zale EL, Plotkin SR, Jordan J, Macklin E, Bakhshaie J (2023). Effect of mind-body skills training on quality of life for geographically diverse adults with neurofibromatosis: a fully remote randomized clinical trial. JAMA Netw Open.

[88] Lester EG, Fishbein NS, Peterson A, Vranceanu AM (2022). Early feasibility testing of a web-based mind-body resiliency program for adults with neurofibromatosis: the NF-web study. PEC Innov.

[89] Grunberg VA, Vranceanu AM (2023). Integrating mind, body, and technology: building virtual psychosocial programs for medical populations. Health Policy Technol.

[90] Doorley JD, Lentz TA, Yeh GY, Wayne PM, Archer KR, Vranceanu AM (2022). Technology-enhanced delivery models to facilitate the implementation of psychologically informed practice for chronic musculoskeletal pain. Phys Ther.

[91] Mace RA, Greenberg J, Lemaster N, Duarte B, Penn T, Kanaya M, Doorley JD, Burris JL, Jacobs CA, Vranceanu AM (2022). Live video mind-body program for patients with knee osteoarthritis, comorbid depression, and obesity: development and feasibility pilot study. JMIR Form Res.

[92] Doorley JD, Mace RA, Popok PJ, Grunberg VA, Ragnhildstveit A, Vranceanu AM (2021). Feasibility randomized controlled trial of a mind-body activity program for older adults with chronic pain and cognitive decline: the virtual "active brains" study. Gerontologist.

[93] Fann JR, Bombardier CH, Vannoy S, Dyer J, Ludman E, Dikmen S, Marshall K, Barber J, Temkin N (2015). Telephone and in-person cognitive behavioral therapy for major depression after traumatic brain injury: a randomized controlled trial. J Neurotrauma.

[94] Exalto LG, Quesenberry CP, Barnes D, Kivipelto M, Biessels GJ, Whitmer RA (2014). Midlife risk score for the prediction of dementia four decades later. Alzheimers Dement.

[95] Kivipelto M, Ngandu T, Laatikainen T, Winblad B, Soininen H, Tuomilehto J (2006). Risk score for the prediction of dementia risk in 20 years among middle aged people: a longitudinal, population-based study. Lancet Neurol.

[96] Mace RA, Gates MV, Popok PJ, Kulich R, Quiroz YT, Vranceanu AM (2021). Feasibility trial of a mind-body activity pain management program for older adults with cognitive decline. Gerontologist.

[97] Pfeffer RI, Kurosaki TT, Harrah CH, Chance JM, Filos S (1982). Measurement of functional activities in older adults in the community. J Gerontol.

[98] Teng E, Becker BW, Woo E, Knopman D, Cummings JL, Lu PH (2010). Utility of the functional activities questionnaire for distinguishing mild cognitive impairment from very mild Alzheimer disease. Alzheimer Dis Assoc Disord.

[99] Alzheimer's Association (2023). 2023 Alzheimer's disease facts and figures. Alzheimers Dement.

[100] Balls-Berry JJE, Babulal GM (2022). Health disparities in dementia. Continuum (Minneap Minn).

[101] Mooldijk SS, Licher S, Wolters FJ (2021). Characterizing demographic, racial, and geographic diversity in dementia research: a systematic review. JAMA Neurol.

[102] Shaw AR, Perales-Puchalt J, Johnson E, Espinoza-Kissell P, Acosta-Rullan M, Frederick S, Lewis A, Chang H, Mahnken J, Vidoni ED (2022). Representation of racial and ethnic minority populations in dementia prevention trials: a systematic review. J Prev Alzheimers Dis.

[103] (2024). Place-based investments: focusing our efforts in the areas of the state experiencing the greatest disparities. Executive Office of Health and Human Services.

[104] Ibrahim S, Sidani S (2014). Strategies to recruit minority persons: a systematic review. J Immigr Minor Health.

[105] Harris PA, Taylor R, Minor BL, Elliott V, Fernandez M, O'Neal L, McLeod L, Delacqua G, Delacqua F, Kirby J, Duda SN, REDCap Consortium (2019). The REDCap consortium: building an international community of software platform partners. J Biomed Inform.

[106] Winckers AN, Mackenbach JD, Compernolle S, Nicolaou M, van der Ploeg HP, De Bourdeaudhuij I, Brug J, Lakerveld J (2015). Educational differences in the validity of self-reported physical activity. BMC Public Health.

[107] Carl J, Grüne E, Popp J, Pfeifer K (2020). Physical activity promotion for apprentices in nursing care and automotive mechatronics-competence counts more than volume. Int J Environ Res Public Health.

[108] Attkisson CC, Zwick R (1982). The client satisfaction questionnaire. Psychometric properties and correlations with service utilization and psychotherapy outcome. Eval Program Plann.

[109] Geisser ME, Clauw DJ, Strand V, Gendreau RM, Palmer R, Williams DA (2010). Contributions of change in clinical status parameters to Patient Global Impression of Change (PGIC) scores among persons with fibromyalgia treated with milnacipran. Pain.

[110] Devilly GJ, Borkovec TD (2000). Psychometric properties of the credibility/expectancy questionnaire. J Behav Ther Exp Psychiatry.

[111] Deckers K, Barbera M, Köhler S, Ngandu T, van Boxtel M, Rusanen M, Laatikainen T, Verhey F, Soininen H, Kivipelto M, Solomon A (2020). Long-term dementia risk prediction by the LIBRA score: a 30-year follow-up of the CAIDE study. Int J Geriatr Psychiatry.

[112] Deckers K, Cadar D, van Boxtel MP, Verhey FR, Steptoe A, Köhler S (2019). Modifiable risk factors explain socioeconomic inequalities in dementia risk: evidence from a population-based prospective cohort study. J Alzheimers Dis.

[113] Deckers K, Nooyens A, van Boxtel M, Verhey F, Verschuren M, Köhler S (2019). Gender and educational differences in the association between lifestyle and cognitive decline over 10 years: the Doetinchem cohort study. J Alzheimers Dis.

[114] Pons A, LaMonica HM, Mowszowski L, Köhler S, Deckers K, Naismith SL (2018). Utility of the LIBRA index in relation to cognitive functioning in a clinical health seeking sample. J Alzheimers Dis.

[115] Schiepers OJ, Köhler S, Deckers K, Irving K, O'Donnell CA, van den Akker M, Verhey FR, Vos SJ, de Vugt ME, van Boxtel MP (2018). Lifestyle for Brain Health (LIBRA): a new model for dementia prevention. Int J Geriatr Psychiatry.

[116] Vos SJ, van Boxtel MP, Schiepers OJ, Deckers K, de Vugt M, Carrière I, Dartigues JF, Peres K, Artero S, Ritchie K, Galluzzo L, Scafato E, Frisoni GB, Huisman M, Comijs HC, Sacuiu SF, Skoog I, Irving K, O'Donnell CA, Verhey FR, Visser PJ, Köhler S (2017). Modifiable risk factors for prevention of dementia in midlife, late life and the oldest-old: validation of the LIBRA index. J Alzheimers Dis.

[117] Sperling RA, Donohue MC, Raman R, Sun CK, Yaari R, Holdridge K, Siemers E, Johnson KA, Aisen PS, A4 Study Team (2020). Association of factors with elevated amyloid burden in clinically normal older individuals. JAMA Neurol.

[118] Li C, Hong Y, Yang X, Zeng X, Ocepek-Welikson K, Eimicke JP, Kong J, Sano M, Zhu C, Neugroschl J, Aloysi A, Cai D, Martin J, Loizos M, Sewell M, Akrivos J, Evans K, Sheppard F, Greenberg J, Ardolino A, Teresi JA (2023). The use of subjective cognitive complaints for detecting mild cognitive impairment in older adults across cultural and linguistic groups: a comparison of the Cognitive Function Instrument to the Montreal Cognitive Assessment. Alzheimers Dement.

[119] Ruthirakuhan M, Wood Alexander M, Cogo-Moreira H, Robinson T, Amariglio R, Buckley R, Sperling R, Swardfager W, Black S, Rabin J (2024). Investigating the factor structure of the preclinical Alzheimer cognitive composite and cognitive function index across racial/ethnic, sex, and Aβ status groups in the A4 study. J Prev Alzheimers Dis.

[120] Amariglio RE, Sikkes SA, Marshall GA, Buckley RF, Gatchel JR, Johnson KA, Rentz DM, Donohue MC, Raman R, Sun CK, Yaari R, Holdridge KC, Sims JR, Grill JD, Aisen PS, Sperling RA (2021). Item-level investigation of participant and study partner report on the cognitive function index from the A4 study screening data. J Prev Alzheimers Dis.

[121] Randolph C, Tierney MC, Mohr E, Chase TN (1998). The repeatable battery for the assessment of neuropsychological status (RBANS): preliminary clinical validity. J Clin Exp Neuropsychol.

[122] Gontkovsky ST, Beatty WW, Mold JW (2004). Repeatable battery for the assessment of neuropsychological status in a normal, geriatric sample. Clin Gerontol.

[123] Stone AA, Broderick JE, Junghaenel DU, Schneider S, Schwartz JE (2016). PROMIS fatigue, pain intensity, pain interference, pain behavior, physical function, depression, anxiety, and anger scales demonstrate ecological validity. J Clin Epidemiol.

[124] Crins MH, van der Wees PJ, Klausch T, van Dulmen SA, Roorda LD, Terwee CB (2018). Psychometric properties of the PROMIS physical function item bank in patients receiving physical therapy. PLoS One.

[125] Amireault S, Godin G, Lacombe J, Sabiston CM (2015). The use of the godin-shephard leisure-time physical activity questionnaire in oncology research: a systematic review. BMC Med Res Methodol.

[126] Amireault S, Godin G (2015). The Godin-Shephard leisure-time physical activity questionnaire: validity evidence supporting its use for classifying healthy adults into active and insufficiently active categories. Percept Mot Skills.

[127] Godin G, Shephard RJ (1985). A simple method to assess exercise behavior in the community. Can J Appl Sport Sci.

[128] vivosmart® 5. Garmin.

[129] Del Pozo Cruz B, Ahmadi M, Naismith SL, Stamatakis E (2022). Association of daily step count and intensity with incident dementia in 78 430 adults living in the UK. JAMA Neurol.

[130] Demeyer H, Burtin C, Hornikx M, Camillo CA, Van Remoortel H, Langer D, Janssens W, Troosters T (2016). The minimal important difference in physical activity in patients with COPD. PLoS One.

[131] Buysse DJ, Reynolds CF, Monk TH, Berman SR, Kupfer DJ (1989). The Pittsburgh Sleep Quality Index: a new instrument for psychiatric practice and research. Psychiatry Res.

[132] Backhaus J, Junghanns K, Broocks A, Riemann D, Hohagen F (2002). Test-retest reliability and validity of the Pittsburgh Sleep Quality Index in primary insomnia. J Psychosom Res.

[133] Beaudreau SA, Spira AP, Stewart A, Kezirian EJ, Lui LY, Ensrud K, Redline S, Ancoli-Israel S, Stone KL, Study of Osteoporotic Fractures (2012). Validation of the Pittsburgh Sleep Quality Index and the Epworth Sleepiness Scale in older black and white women. Sleep Med.

[134] Lysen TS, Luik AI, Ikram MK, Tiemeier H, Ikram MA (2020). Actigraphy-estimated sleep and 24-hour activity rhythms and the risk of dementia. Alzheimers Dement.

[135] Papaconstantinou E, Cancelliere C, Verville L, Wong JJ, Connell G, Yu H, Shearer H, Timperley C, Chung C, Porter BJ, Myrtos D, Barrigar M, Taylor-Vaisey A (2021). Effectiveness of non-pharmacological interventions on sleep characteristics among adults with musculoskeletal pain and a comorbid sleep problem: a systematic review. Chiropr Man Therap.

[136] Cerwinske LA, Rasmussen HE, Lipson S, Volgman AS, Tangney CC (2017). Evaluation of a dietary screener: the Mediterranean eating pattern for Americans tool. J Hum Nutr Diet.

[137] Pilkonis PA, Yu L, Dodds NE, Johnston KL, Lawrence SM, Daley DC (2016). Validation of the alcohol use item banks from the Patient-Reported Outcomes Measurement Information System (PROMIS). Drug Alcohol Depend.

[138] Gibbons LE, Fredericksen R, Merrill JO, McCaul ME, Chander G, Hutton H, Lober WB, Mathews WC, Mayer K, Burkholder G, Willig JH, Mugavero MJ, Saag MS, Kitahata MM, Edwards TC, Patrick DL, Crane HM, Crane PK (2016). Suitability of the PROMIS alcohol use short form for screening in a HIV clinical care setting. Drug Alcohol Depend.

[139] (2024). Behavioral risk factor surveillance system survey data. Centers for Disease Control and Prevention (CDC).

[140] Deborah A (2023). Developing the Patient-Reported Outcomes Measurement Information System (PROMIS). Med Care.

[141] Carlozzi NE, Ianni PA, Lange RT, Brickell TA, Kallen MA, Hahn EA, French LM, Cella D, Miner JA, Tulsky DS (2019). Understanding health-related quality of life of caregivers of civilians and service members/veterans with traumatic brain injury: establishing the reliability and validity of PROMIS social health measures. Arch Phys Med Rehabil.

[142] Marti CN, Choi NG (2022). Measuring social engagement among low-income, depressed homebound older adults: validation of the social engagement and activities questionnaire. Clin Gerontol.

[143] Bode RK, Hahn EA, DeVellis R, Cella D, Patient-Reported Outcomes Measurement Information System Social Domain Working Group (2010). Measuring participation: the Patient-Reported Outcomes Measurement Information System experience. Arch Phys Med Rehabil.

[144] Hahn EA, Devellis RF, Bode RK, Garcia SF, Castel LD, Eisen SV, Bosworth HB, Heinemann AW, Rothrock N, Cella D, PROMIS Cooperative Group (2010). Measuring social health in the patient-reported outcomes measurement information system (PROMIS): item bank development and testing. Qual Life Res.

[145] Garrett DD, Grady CL, Hasher L (2010). Everyday memory compensation: the impact of cognitive reserve, subjective memory, and stress. Psychol Aging.

[146] Geda YE, Topazian HM, Lewis RA, Roberts RO, Knopman DS, Pankratz VS, Christianson TJ, Boeve BF, Tangalos EG, Ivnik RJ, Petersen RC (2011). Engaging in cognitive activities, aging, and mild cognitive impairment: a population-based study. J Neuropsychiatry Clin Neurosci.

[147] Verghese J, Lipton RB, Katz MJ, Hall CB, Derby CA, Kuslansky G, Ambrose AF, Sliwinski M, Buschke H (2003). Leisure activities and the risk of dementia in the elderly. N Engl J Med.

[148] Wilson RS, Bennett DA, Beckett LA, Morris MC, Gilley DW, Bienias JL, Scherr PA, Evans DA (1999). Cognitive activity in older persons from a geographically defined population. J Gerontol B Psychol Sci Soc Sci.

[149] Pilkonis PA, Choi SW, Reise SP, Stover AM, Riley WT, Cella D, PROMIS Cooperative Group (2011). Item banks for measuring emotional distress from the Patient-Reported Outcomes Measurement Information System (PROMIS®): depression, anxiety, and anger. Assessment.

[150] Teresi JA, Ocepek-Welikson K, Kleinman M, Ramirez M, Kim G (2016). Psychometric properties and performance of the patient reported outcomes measurement information system (PROMIS) depression short forms in ethnically diverse groups. Psychol Test Assess Model.

[151] Schalet BD, Pilkonis PA, Yu L, Dodds N, Johnston KL, Yount S, Riley W, Cella D (2016). Clinical validity of PROMIS depression, anxiety, and anger across diverse clinical samples. J Clin Epidemiol.

[152] Sunderland M, Batterham P, Calear A, Carragher N (2018). Validity of the PROMIS depression and anxiety common metrics in an online sample of Australian adults. Qual Life Res.

[153] Li MJ, Black DS, Garland EL (2016). The Applied Mindfulness Process Scale (AMPS): a process measure for evaluating mindfulness-based interventions. Pers Individ Dif.

[154] Gross JJ, John OP (2003). Individual differences in two emotion regulation processes: implications for affect, relationships, and well-being. J Pers Soc Psychol.

[155] Brady B, Kneebone II, Bailey PE (2019). Validation of the emotion regulation questionnaire in older community-dwelling adults. Br J Clin Psychol.

[156] Preece DA, Becerra R, Robinson K, Gross JJ (2020). The emotion regulation questionnaire: psychometric properties in general community samples. J Pers Assess.

[157] Gabrys R, Tabri N, Anisman H, Matheson K (2018). Cognitive control and flexibility in the context of stress and depressive symptoms: the cognitive control and flexibility questionnaire. Front Psychol.

[158] Kim S, Sargent-Cox K, Cherbuin N, Anstey KJ (2014). Development of the motivation to change lifestyle and health behaviours for dementia risk reduction scale. Dement Geriatr Cogn Dis Extra.

[159] Papp KV, Samaroo AH, Chou HL, Buckley RF, Rentz D, Sperling RA, Amariglio R (2020). Repeated memory‐based assessments: implications for clinical trials and practice. Alzheimers Dement.

[160] Weizenbaum EL, Soberanes D, Hsieh S, Molinare CP, Buckley RF, Betensky RA, Properzi MJ, Marshall GA, Rentz DM, Johnson KA, Sperling RA, Amariglio RE, Papp KV (2024). Capturing learning curves with the multiday Boston Remote Assessment of Neurocognitive Health (BRANCH): feasibility, reliability, and validity. Neuropsychology.

[161] Garland EL, Hanley AW, Nakamura Y, Barrett JW, Baker AK, Reese SE, Riquino MR, Froeliger B, Donaldson GW (2022). Mindfulness-oriented recovery enhancement vs supportive group therapy for co-occurring opioid misuse and chronic pain in primary care: a randomized clinical trial. JAMA Intern Med.

[162] Kratz AL, Murphy SL, Braley TJ (2017). Pain, fatigue, and cognitive symptoms are temporally associated within but not across days in multiple sclerosis. Arch Phys Med Rehabil.

[163] Kratz AL, Murphy SL, Braley TJ (2017). Ecological momentary assessment of pain, fatigue, depressive, and cognitive symptoms reveals significant daily variability in multiple sclerosis. Arch Phys Med Rehabil.

[164] Davalos DB, Luxton I, Thaut M, Cross JE (2019). B Sharp-The cognitive effects of a pilot community music program for people with dementia-related disorders. Alzheimers Dement (N Y).

[165] Gray M, Madero EN, Gills JL, Paulson S, Jones MD, Campitelli A, Myers J, Bott NT, Glenn JM (2022). Intervention for a digital, cognitive, multi-domain Alzheimer risk velocity study: protocol for a randomized controlled trial. JMIR Res Protoc.

[166] Birberg Thornberg U, Andersson A, Lindh M, Hellgren L, Divanoglou A, Levi R (2023). Neurocognitive deficits in COVID-19 patients five months after discharge from hospital. Neuropsychol Rehabil.

[167] Duff K, Beglinger LJ, Schoenberg MR, Patton DE, Mold J, Scott JG, Adams RL (2005). Test-retest stability and practice effects of the RBANS in a community dwelling elderly sample. J Clin Exp Neuropsychol.

[168] Patton DE, Duff K, Schoenberg MR, Mold J, Scott JG, Adams RL (2003). Performance of cognitively normal African Americans on the RBANS in community dwelling older adults. Clin Neuropsychol.

[169] Miller TW, Wood JA, Information Resources Management Association - (2011). Telepractice. Clinical Technologies: Concepts, Methodologies, Tools and Applications.

[170] Foster JI, Williams KL, Timmer BH, Brauer SG (2022). Concurrent validity of the Garmin Vivofit®4 to accurately record step count in older adults in challenging environments. J Aging Phys Act.

[171] Larsen RT, Korfitsen CB, Juhl CB, Andersen HB, Christensen J, Langberg H (2020). The MIPAM trial: a 12-week intervention with motivational interviewing and physical activity monitoring to enhance the daily amount of physical activity in community-dwelling older adults - a study protocol for a randomized controlled trial. BMC Geriatr.

[172] Gaz DV, Rieck TM, Peterson NW, Ferguson JA, Schroeder DR, Dunfee HA, Henderzahs-Mason JM, Hagen PT (2018). Determining the validity and accuracy of multiple activity-tracking devices in controlled and free-walking conditions. Am J Health Promot.

[173] Höchsmann C, Knaier R, Eymann J, Hintermann J, Infanger D, Schmidt-Trucksäss A (2018). Validity of activity trackers, smartphones, and phone applications to measure steps in various walking conditions. Scand J Med Sci Sports.

[174] Evenson KR, Spade CL (2020). Review of validity and reliability of Garmin activity trackers. J Meas Phys Behav.

[175] Huhn S, Axt M, Gunga HC, Maggioni MA, Munga S, Obor D, Sié A, Boudo V, Bunker A, Sauerborn R, Bärnighausen T, Barteit S (2022). The impact of wearable technologies in health research: scoping review. JMIR Mhealth Uhealth.

[176] Kim M (2015). Association between objectively measured sleep quality and obesity in community-dwelling adults aged 80 years or older: a cross-sectional study. J Korean Med Sci.

[177] Buchman AS, Boyle PA, Yu L, Shah RC, Wilson RS, Bennett DA (2012). Total daily physical activity and the risk of AD and cognitive decline in older adults. Neurology.

[178] Buchman AS, Wang T, Oveisgharan S, Zammit AR, Yu L, Li P, Hu K, Hausdorff JM, Lim AS, Bennett DA (2023). Correlates of person-specific rates of change in sensor-derived physical activity metrics of daily living in the rush memory and aging project. Sensors (Basel).

[179] Chung J, Brakey HR, Reeder B, Myers O, Demiris G (2023). Community-dwelling older adults' acceptance of smartwatches for health and location tracking. Int J Older People Nurs.

[180] Troiano RP, Berrigan D, Dodd KW, Mâsse LC, Tilert T, McDowell M (2008). Physical activity in the United States measured by accelerometer. Med Sci Sports Exerc.

[181] Chan A, Chan D, Lee H, Ng CC, Yeo AH (2022). Reporting adherence, validity and physical activity measures of wearable activity trackers in medical research: a systematic review. Int J Med Inform.

[182] Song J, Semanik P, Sharma L, Chang RW, Hochberg MC, Mysiw WJ, Bathon JM, Eaton CB, Jackson R, Kwoh CK, Nevitt M, Dunlop DD (2010). Assessing physical activity in persons with knee osteoarthritis using accelerometers: data from the osteoarthritis initiative. Arthritis Care Res (Hoboken).

[183] Degroote L, De Bourdeaudhuij I, Verloigne M, Poppe L, Crombez G (2018). The accuracy of smart devices for measuring physical activity in daily life: validation study. JMIR Mhealth Uhealth.

[184] Fitabase. Small Steps Labs LLC.

[185] Cole RJ, Kripke DF, Gruen W, Mullaney DJ, Gillin JC (1992). Automatic sleep/wake identification from wrist activity. Sleep.

[186] Cellini N, Buman MP, McDevitt EA, Ricker AA, Mednick SC (2013). Direct comparison of two actigraphy devices with polysomnographically recorded naps in healthy young adults. Chronobiol Int.

[187] Papp KV, Samaroo A, Chou HC, Buckley R, Schneider OR, Hsieh S, Soberanes D, Quiroz Y, Properzi M, Schultz A, García-Magariño I, Marshall GA, Burke JG, Kumar R, Snyder N, Johnson K, Rentz DM, Sperling RA, Amariglio RE (2021). Unsupervised mobile cognitive testing for use in preclinical Alzheimer's disease. Alzheimers Dement (Amst).

[188] Papp KV, Jutten RJ, Soberanes D, Weizenbaum E, Hsieh S, Molinare C, Buckley R, Betensky RA, Marshall GA, Johnson KA, Rentz DM, Sperling R, Amariglio RE (2024). Early detection of amyloid-related changes in memory among cognitively unimpaired older adults with daily digital testing. Ann Neurol.

[189] Gold D, Stockwood J, Boulos K, Kasha S, Vyshedskiy A, deTorres L, Ostrovsky S, Durakovic D, Savchenko A, Piryatinsky I (2022). The Boston cognitive assessment: psychometric foundations of a self-administered measure of global cognition. Clin Neuropsychol.

[190] Nielsen L, Riddle M, King JW, Aklin WM, Chen W, Clark D, Collier E, Czajkowski S, Esposito L, Ferrer R, Green P, Hunter C, Kehl K, King R, Onken L, Simmons JM, Stoeckel L, Stoney C, Tully L, Weber W, NIH Science of Behavior Change Implementation Team (2018). The NIH science of behavior change program: transforming the science through a focus on mechanisms of change. Behav Res Ther.

[191] Reuter-Lorenz PA, Park DC (2014). How does it STAC up? Revisiting the scaffolding theory of aging and cognition. Neuropsychol Rev.

[192] Mintzer J, Donovan KA, Kindy AZ, Lock SL, Chura LR, Barracca N (2019). Lifestyle choices and brain health. Front Med (Lausanne).

[193] Sabayan B, Sorond F (2017). Reducing risk of dementia in older age. JAMA.

[194] Mace RA, Gates MV, Bullard B, Lester EG, Silverman IH, Quiroz YT, Vranceanu AM (2021). Development of a novel mind-body activity and pain management program for older adults with cognitive decline. Gerontologist.

[195] Hobbis IC, Sutton S (2005). Are techniques used in cognitive behaviour therapy applicable to behaviour change interventions based on the theory of planned behaviour?. J Health Psychol.

[196] Doran GT (1981). There's a S.M.A.R.T. way to write managements's goals and objectives. Manage Rev.

[197] Hooker JE, Brewer JR, McDermott KA, Kanaya M, Somers TJ, Keefe F, Kelleher S, Fisher HM, Burns J, Jeddi RW, Kulich R, Polykoff G, Parker RA, Greenberg J, Vranceanu A, THRIVE Study Team (2024). Improving multimodal physical function in adults with heterogeneous chronic pain; protocol for a multisite feasibility RCT. Contemp Clin Trials.

[198] Greenberg J, Popok PJ, Lin A, Kulich RJ, James P, Macklin EA, Millstein RA, Edwards RR, Vranceanu AM (2020). A mind-body physical activity program for chronic pain with or without a digital monitoring device: proof-of-concept feasibility randomized controlled trial. JMIR Form Res.

[199] Carter S, Greenberg J, Funes CJ, Macklin EA, Vranceanu AM (2021). Effects of a mind-body program on symptoms of depression and perceived stress among adults with neurofibromatosis type 2 who are deaf: a live-video randomized controlled trial. Complement Ther Med.

[200] Greenberg J, Singh T, Iverson GL, Silverberg ND, Macklin EA, Parker RA, Giacino JT, Yeh GY, Vranceanu AM (2021). A live video mind-body treatment to prevent persistent symptoms following mild traumatic brain injury: protocol for a mixed methods study. JMIR Res Protoc.

[201] Greenberg J, Carter S, Lester E, Funes CJ, Macklin EA, Plotkin S, Vranceanu AM (2019). Cultivating resiliency in patients with neurofibromatosis 2 who are deafened or have severe hearing loss: a live‑video randomized control trial. J Neurooncol.

[202] RStudio: integrated development environment for R. RStudio Team.

[203] R Core Team R: a language and environment for statistical computing. R Foundation for Statistical Computing.

[204] Cohen J (1988). Statistical Power Analysis for the Behavioral Sciences. 2nd edition.

[205] Proctor E, Silmere H, Raghavan R, Hovmand P, Aarons G, Bunger A, Griffey R, Hensley M (2011). Outcomes for implementation research: conceptual distinctions, measurement challenges, and research agenda. Adm Policy Ment Health.

[206] Damschroder LJ, Aron DC, Keith RE, Kirsh SR, Alexander JA, Lowery JC (2009). Fostering implementation of health services research findings into practice: a consolidated framework for advancing implementation science. Implement Sci.

[207] Powell BJ, Waltz TJ, Chinman MJ, Damschroder LJ, Smith JL, Matthieu MM, Proctor EK, Kirchner JE (2015). A refined compilation of implementation strategies: results from the Expert Recommendations for Implementing Change (ERIC) project. Implement Sci.

[208] Stokols D, Allen J, Bellingham RL (2016). The social ecology of health promotion: implications for research and practice. Am J Health Promot.

[209] Aminzadeh F, Molnar FJ, Dalziel WB, Ayotte D (2012). A review of barriers and enablers to diagnosis and management of persons with dementia in primary care. Can Geriatr J.

[210] Stephan A, Bieber A, Hopper L, Joyce R, Irving K, Zanetti O, Portolani E, Kerpershoek L, Verhey F, de Vugt M, Wolfs C, Eriksen S, Røsvik J, Marques MJ, Gonçalves-Pereira M, Sjölund B, Jelley H, Woods B, Meyer G, Actifcare Consortium (2018). Barriers and facilitators to the access to and use of formal dementia care: findings of a focus group study with people with dementia, informal carers and health and social care professionals in eight European countries. BMC Geriatr.

[211] Kulmala J, Rosenberg A, Ngandu T, Hemiö K, Tenkula T, Hyytiä A, Vienola M, Huhtamäki-Kuoppala M, Saarinen A, Korkki S, Laatikainen T, Solomon A, Kivipelto M (2021). Facilitators and barriers to implementing lifestyle intervention programme to prevent cognitive decline. Eur J Public Health.

[212] Rosenberg A, Coley N, Soulier A, Kulmala J, Soininen H, Andrieu S, Kivipelto M, Barbera M, MIND-ADHATICE groups (2020). Experiences of dementia and attitude towards prevention: a qualitative study among older adults participating in a prevention trial. BMC Geriatr.

[213] Mace RA, Mattos MK, Vranceanu AM (2022). Older adults can use technology: why healthcare professionals must overcome ageism in digital health. Transl Behav Med.

[214] Coley N, Coniasse-Brioude D, Igier V, Fournier T, Poulain JP, Andrieu S, ACCEPT study group (2021). Disparities in the participation and adherence of older adults in lifestyle-based multidomain dementia prevention and the motivational role of perceived disease risk and intervention benefits: an observational ancillary study to a randomised controlled trial. Alzheimers Res Ther.

[215] James T, Mukadam N, Sommerlad A, Guerra Ceballos S, Livingston G (2021). Culturally tailored therapeutic interventions for people affected by dementia: a systematic review and new conceptual model. Lancet Healthy Longev.

[216] Savold J, Cole M, Thorpe RJ (2023). Barriers and solutions to Alzheimer's disease clinical trial participation for Black Americans. Alzheimers Dement (N Y).

