# Supplementary material for: A Mindfulness-Based Lifestyle Intervention for Dementia Risk Reduction: Protocol for the My Healthy Brain Feasibility Randomized Controlled Trial
Source: JMIR Res Protoc. 2024 Nov 21;13:e64149. doi: 10.2196/64149 (PMC11621724; doi:10.2196/64149)
Supplement: Multimedia Appendix 1 [file resprot_v13i1e64149_app1.docx]

| **Category** | **Protocol Changes and Strategies** | **Rationales** |
| --- | --- | --- |
| Recruitment | Outreach in community organizations, senior centers, and community health clinics  Disseminate recruitment materials via social media, newsletters, and online research platforms | Our institution primarily servers white and highly affluent patients  Increase recruitment and enrollment of diverse older adults  Address disparities in AD/ADRD prevention |
|  | Delivered educational presentations to the public (e.g., talks, Q&A, interactive games) | Increase public knowledge and awareness of AD/ADRD and modifiable lifestyle risk factors |
|  | Engaged clinic leadership and champions  Created a referral toolkit for providers (e.g., cheat sheet with eligibility criteria and study selling points) | Promote institutional commitment to the study  Improve quality and timeliness of provider referrals |
| Enrollment | Streamlined enrollment process via an online eligibility self-screener | Reduce participant burden |
|  | Increased trial compensation | Reduce financial barriers to participation |
|  | Provided list of behavioral health resources developed by our center’s Community Engagement Core | Provide alternative resources and services for older adults who are ineligible or need higher levels of care |
|  | Translated participant resources for people with memory related concerns into Spanish (i.e., bilingual pamphlet with no-cost resources) | Address informational disparities in AD/ADRD prevention among Spanish-speaking older adults |
| Technical Support | Provided iPads for participants without smartphones and/or computers | Reduce technology access barriers |
|  | Developed at step-by-step user guide for the iPad, Garmin Vívosmart® 5 watch, and Garmin Connect™ app | Promote digital health readiness and literacy among older adults |
|  | Optimize iPad and Garmin Vívosmart® 5 displays (limiting number of apps, accessibility mode, stylus) | Ensure that study technologies are age-friendly |
|  | Implemented 1:1 coaching for technology (i.e., how to use Zoom, how to use the Garmin Garmin Vívosmart® 5)  Check in with participants each week for additional technological support as needed | Provide consistent and reliable support throughout the RCT |
|  | Involved caregivers, friends, and family for additional support when needed | Involve supports that are trusted and available to reinforce technology use |
|  | Added instructions and adherence strategies for the Garmin Vívosmart® 5 to the program manuals | Clarify watch wear and data collection expectations |
| Interventions | Revised manual to 6^th^ grade reading level  Increased the use of visual aids and lay language to convey program concepts (e.g., mindfulness, brain health, neuroplasticity, cognitive reserve)  Adopted terminology preferred by older adults (e.g., memory-related problems instead of cognitive decline).  Integrated shortcuts to website and program materials in the manual (QR codes, bookmarks, etc.)  Streamlined homework checklists  Removed content and skills not related to intervention targets | Manual improvements based on feedback from participants, healthcare professionals, our multidisciplinary team, and “lessons learned” from prior studies |
|  | Developed multimedia educational materials for the website (e.g., “what is mindfulness?” video)  Added “Bonus Meditations” to encourage the application of informal mindfulness practices (i.e., mindful walking) to daily living  Created spaces for self-reflection in the manual (e.g., goals, questions)  Developed mindfulness recordings in both male and female voices  Aligned intervention components with individual patient motivations | Improve uptake of mindfulness and lifestyle behavior modification |
|  | Included affordable options for lifestyle activities (i.e., walking and community fitness classes, canned and frozen food) | Acceptability and appropriateness of lifestyle intervention for older adults from lower socioeconomic backgrounds |
